# The Causal Relationship Between Neurotrophic Factors and Delirium: A Mendelian Randomization Study

**DOI:** 10.1002/brb3.70494

**Published:** 2025-05-05

**Authors:** Han Wu, Ruilai Jiang, Xiaocheng Huang, Xiaogang Hu

**Affiliations:** ^1^ Department of Geriatrics the Second People's Hospital of Lishui Lishui China; ^2^ Zhejiang Chinese Medical University Hangzhou China

**Keywords:** delirium, glial cell‐derived neurotrophic factor, Mendelian randomization, neurotrophic factors

## Abstract

**Background:**

Several observational studies have revealed that different neurotrophic factors (NTFs) are associated with delirium, yet the direction and magnitude of the causal association remain poorly understood. Herein, we performed a two‐sample Mendelian randomization (MR) analysis to investigate the causal relationship between these factors and delirium.

**Methods:**

GWAS data for delirium were sourced from the FINN10 database; GWAS data for risk factors (protein kinase C‐binding protein NELL1, neurotrophin‐3, neurotrophin‐4, brain‐derived neurotrophic factor levels, nerve growth factor, ciliary neurotrophic factor, and glial cell‐derived neurotrophic factor levels) were from the IEU Open GWAS. Inverse‐variance weighted (IVW) was used as a primary analysis. MR‐Egger, weighted median (WM), and weighted model were applied to validate the robustness of the results. The MR‐Egger regression method was used to explore the presence of horizontal pleiotropy, and the MR pleiotropy residual sum, and outlier (MR‐PRESSO) method was applied to detect potential outliers. Cochran's Q test assessed heterogeneity among instrumental variables (IVs). The leave‐one‐out (LOO) method was used to enhance the precision and veracity of our findings.

**Results:**

IVW analyses revealed no association between risk factors and delirium. MR Egger, WM, and the weighted mode approach further confirmed these data. MR‐Egger regression analysis confirmed the absence of directional pleiotropy in our analysis. Heterogeneity and sensitivity analyses showed reliable results.

**Conclusion:**

No association between other factors and delirium was identified; however, further research is needed to determine if these results apply to other races. Also, advances in molecular biology and epigenetics may shed light on this topic.

## Graphical abstract

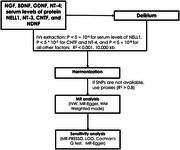

## 1 Introduction

Delirium is a severe neuropsychiatric condition characterized by acute attention, awareness, and cognition changes. It often manifests as altered arousal, delusions and hallucinations, and mood variations (Wilson et al. 2020). Additional symptoms include psychomotor disturbances, disrupted sleep‐wake cycle, memory impairment, emotional variability, and disorientation (Wilson et al. 2020). Older individuals, especially those with preexisting conditions, seem most susceptible to this condition (Bellelli et al. 2021), and according to available data, delirium prevalence rates range from 10%–22% at hospital admission and 10%–38% during hospital stay (Al Farsi et al. 2023). Although the pathophysiology of delirium has not yet been completely elucidated, inflammation, hypoxia, and oxidative stress are all considered relevant factors due to their implication in the increased brain exposure to toxins and a hypocholinergic‐hyperdopaminergic state (Thom et al. 2019). Inflammation can deteriorate the overall physiological state, impair brain function, and increase blood‐brain barrier permeability. Vulnerability to circulating deliriogenic medications, endogenous toxins, and proinflammatory cytokines may directly cause or promote delirium (Bellelli et al. 2021).

Neurotrophic factors (NTFs) are a group of proteins that regulate the proliferation, survival, migration, and differentiation of cells in the nervous system (Castren et al. 2007). Studies have revealed that different NTFs, particularly glial cell‐derived neurotrophic factor (GDNF), ciliary neurotrophic factor (CNTF), brain‐derived neurotrophic factor (BDNF), nerve growth factor (NGF), neurotrophin‐3 (NT‐3), and NT‐4/5 exert a regenerative role in different animal models and patients with neuroinflammatory, and neurodegenerative diseases (Tian et al. 2012, Palasz et al. 2023). Accordingly, NTFs have gained increasing interest as a potential therapy for neurogenerative diseases (El Ouaamari et al. 2023).

BDNF has been the most extensively studied NTF regarding its involvement in delirium. In fact, studies have investigated its role in delirium, with inconsistent results being reported. E.g., a study that included patients treated at intensive care units found significantly higher levels of BDNF in patients with delirium than in those without (Grandi et al. 2011). However, a different study found no significant associations in BDNF levels between older medical delirious and non‐delirious inpatients (Williams et al. 2017). Yet, observational studies do not benefit from random treatment assignment, and therefore, uncontrolled confounding constitutes a potentially serious validity concern. Also, the direction and magnitude of the causal association between these factors and delirium remain unclear.

MR analyses are based on identifying variable natural phenomena (known generically as IVs) used in the statistical analysis to adjust for possible confounding factors in the research study (Sanderson et al. 2022). This analytical research method uses genetic variation associated with specific exposures of interest to explore the causal relationships between potentially changeable risk factors and health outcomes in observational data (Sanderson et al. 2022). Considering that the distribution of genetic variants is approximately random and occurs at conception, this type of analytical approach is considered to be less prone to confounding, reverse causation, measurement error, and other biases that can potentially limit the validity of observational studies, thus permitting inferences to be made about causality (Richmond and Davey Smith 2022).

The aim of this study was to investigate the causal relationship between NTFs (namely protein kinase C‐binding protein NELL1, NT‐3, NT‐4 levels, and BDNF, NGF, CNTF, and GDNF) and delirium.

## 2 Materials and Methods

The quality of the study was assessed using the strengthening the reporting of observational studies in Epidemiology using Mendelian randomization (STROBE‐MR) guidelines (Skrivankova et al. 2021).

### 2.1 Data Source

Data utilized in this study were sourced from public databases; therefore, ethical approval was not required. All populations analyzed were of European ancestry. GWAS data for delirium were sourced from the FINN10 database that included 3371 patients with delirium and 3,88,560 controls. GWAS data for risk factors (namely protein kinase C‐binding protein NELL1, NT‐3, NT‐4 levels, and serum levels of NELL1, BDNF, NGF, CNTF, and GDNF) were from the IEU Open GWAS Project; more data regarding data sources are shown in Table S1.

### 2.2 Instrumental Variables (IVs) Selection and MR Analysis

**FIGURE 1:**
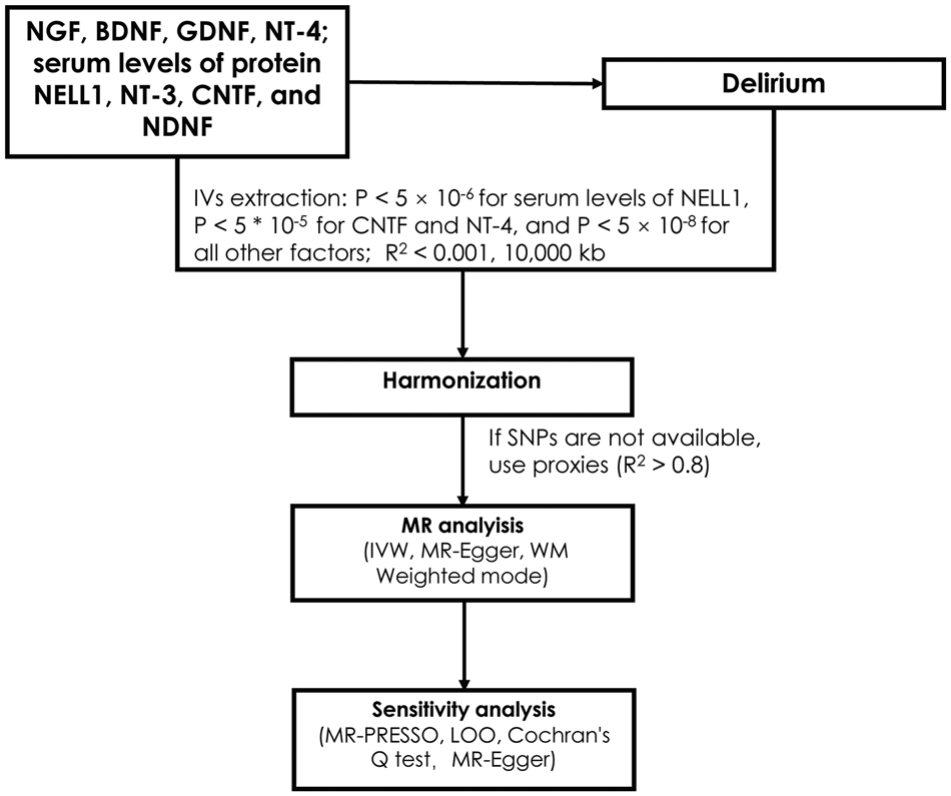
Overview of MR analysis. SNPs: single nucleotide polymorphisms; inverse‐variance weighted (IVW); weighted median (WM); leave‐one‐out (LOO); glial cell‐derived neurotrophic factor (GDNF); ciliary neurotrophic factor (CNTF); brain‐derived neurotrophic factor (BDNF); nerve growth factor (NGF); neurotrophin‐3 (NT‐3); neurotrophin‐4 (NT‐4); brain‐derived neurotrophic factor (BDNF); nerve growth factor (NGF); neural epidermal growth factor‐like 1 protein NELL1.

MR analysis was based on three assumptions (Figure 1) (Martens et al. 2006): (1) it was not associated with the outcomes due to confounding pathways; (2) it did not affect the outcome; and (3) it was associated with the exposure. SNPs were selected as IVs based on the following criteria (Skrivankova et al. 2021): (i) SNPs significantly associated with risk factors were selected using P < 5 * 10^−8;^ due to the paucity of SNPs related to CNTF, NT‐4, and serum levels of protein NELL1 passing the initial screening threshold, the criteria were relaxed to include those with P < 5 * 10^−6^ for serum levels of protein NELL1 and P < 5 * 10^−5^ for CNTF and NT‐4; (ii) SNPs with a minimum minor allele frequency (MAF) > 0.01 were screened; (iii) R^2^ < 0.001 and window size = 10,000 kb were used for linkage disequilibrium (LD) between SNPs; (iv) when the selected IVs were not present in the summary data of the outcome, we searched for SNPs with high LD (R^2^ > 0.8) with the IVs as proxy SNPs to replace the existing ones (Skrivankova et al. 2021); and (v) the F‐value for each SNP in the IV was calculated to assess IV strength, excluding potential weak instrument bias between the IV and exposure factors, using the following formula: F = R^2^ * (N‐2)/(1‐R^2^), where R^2^ represented the proportion of exposure variance explained by the SNP in the IV. The requirement for the F‐value was > 10.

### 2.3 Evaluation of Robustness in the Identified Associations

IVW was primarily used to estimate the causal relationship between the exposure and outcome by calculating the odds ratio (OR) and 95% confidence interval (CI). The weighted model, weighted median (WM), and MR‐Egger were further used to validate the results. The MR‐Egger method takes into account the presence of an intercept term and provides accurate causal effect estimates even when there is directional pleiotropy (Burgess and Thompson 2017); the weighted median method assumes that at least half of the IVs are valid and examines the causal relationship between the exposure and the outcome.

The MR‐Egger regression method was used to explore the presence of horizontal pleiotropy (Burgess and Thompson 2017), and the MR‐PRESSO method was applied to detect potential outliers. Cochran's Q test assessed heterogeneity among IVs. Sensitivity analyses were performed using the leave‐one‐out (LOO) method to enhance our findings’ precision and veracity; this method enables the assessment of whether or not any causal effect estimate is being driven by one SNP independently of all other SNPs being used as IVs by comparing the original causal effect estimate (i.e., with all SNPs included in the model) to estimates where each SNP has been removed (Burgess et al. 2019). All analyses were performed using R 4.0.5 software, incorporating packages such as TwoSampleMR and MR‐PRESSO. Visualizations were achieved through scatter plots and sensitivity analysis graphs.

## 3 Results

Relevant IVs were selected after assessing the causal association between exposure factors (namely NGF, BDNF, GDNF, NT‐4, serum levels of NELL1, NT‐3, CNTF, and NDNF) and outcomes (delirium). Initially, 6, 22, 31, 9, 12, 24, 22, 22, and 10 IVs were identified when selecting serum levels of protein NELL1, protein kinase C‐binding protein NELL1, serum levels of BDNF, serum levels of NGF, serum levels of NDNF, CNTF levels, GDNF levels, NT‐3 levels, and NT‐4 levels, respectively. All F values of SNPs exceed 10, as shown in Table S2. Detailed information regarding IV screening is shown in Table S3 .

IVW analyses revealed no association between risk factors and delirium [all P > 0.05, Table 1, Figure S1–S9]. These data were further confirmed by MR Egger, WM, and the weighted mode approach, all showing P > 0.05 (Table 1). MR‐Egger regression confirmed the absence of horizontal pleiotropy in our analysis, except for GDNF (egger_intercept: ‐0.048, P = 0.023) (Table 2), which suggests that genetic variants associated with GDNF may be associated with multiple phenotypes. Moreover, no heterogeneity was found (Table 2).

**TABLE 1:** Casual effect of genetically predicted NTFs on delirium.

Outcome	Exposure	Methods	N SNP	P	OR (95%CI)
Delirium	NGF	IVW	9	0.188	0.894 (0.757 ‐ 1.056)
Delirium	NGF	MR Egger	9	0.099	0.741 (0.544 ‐ 1.009)
Delirium	NGF	WM	9	0.074	0.819 (0.658 ‐ 1.019)
Delirium	NGF	Weighted mode	9	0.101	0.811 (0.65 ‐ 1.012)
Delirium	BDNF	IVW	29	0.971	0.998 (0.907 ‐ 1.099)
Delirium	BDNF	MR Egger	29	0.450	0.919 (0.74 ‐ 1.141)
Delirium	BDNF	WM	29	0.327	1.075 (0.93 ‐ 1.242)
Delirium	BDNF	Weighted mode	29	0.515	1.066 (0.882 ‐ 1.289)
Delirium	GDNF	IVW	18	0.518	0.949 (0.81 ‐ 1.112)
Delirium	GDNF	MR Egger	18	0.128	1.224 (0.956 ‐ 1.567)
Delirium	GDNF	WM	18	0.751	1.034 (0.842 ‐ 1.269)
Delirium	GDNF	Weighted mode	18	0.655	1.047 (0.859 ‐ 1.276)
Delirium	NT‐4	IVW	10	0.551	1.034 (0.927 ‐ 1.153)
Delirium	NT‐4	MR Egger	10	0.392	1.217 (0.796 ‐ 1.859)
Delirium	NT‐4	WM	10	0.981	0.998 (0.882 ‐ 1.131)
Delirium	NT‐4	Weighted mode	10	0.801	0.978 (0.824 ‐ 1.16)
Delirium	Serum levels of NELL1	IVW	6	0.231	1.066 (0.96 ‐ 1.185)
Delirium	Serum levels of NELL1	MR Egger	6	0.353	1.084 (0.932 ‐ 1.261)
Delirium	Serum levels of NELL1	WM	6	0.221	1.077 (0.956 ‐ 1.212)
Delirium	Serum levels of NELL1	Weighted mode	6	0.289	1.08 (0.951 ‐ 1.226)
Delirium	NT‐3	IVW	20	0.160	0.882 (0.741 ‐ 1.05)
Delirium	NT‐3	MR Egger	20	0.344	0.835 (0.581 ‐ 1.201)
Delirium	NT‐3	WM	20	0.872	0.98 (0.767 ‐ 1.253)
Delirium	NT‐3	Weighted mode	20	0.994	1.001 (0.685 ‐ 1.463)
Delirium	CNTF	IVW	24	0.614	0.987 (0.936 ‐ 1.04)
Delirium	CNTF	MR Egger	24	0.963	1.004 (0.835 ‐ 1.208)
Delirium	CNTF	WM	24	0.177	0.952 (0.887 ‐ 1.022)
Delirium	CNTF	Weighted mode	24	0.334	0.936 (0.822 ‐ 1.067)
Delirium	NDNF	IVW	12	0.572	1.056 (0.874 ‐ 1.275)
Delirium	NDNF	MR Egger	12	0.716	0.897 (0.507 ‐ 1.587)
Delirium	NDNF	WM	12	0.486	1.079 (0.871 ‐ 1.336)
Delirium	NDNF	Weighted mode	12	0.466	1.111 (0.845 ‐ 1.462)
Delirium	Protein kinase C‐binding protein NELL1	IVW	22	0.510	1.022 (0.959 ‐ 1.089)
Delirium	Protein kinase C‐binding protein NELL1	MR Egger	22	0.262	1.062 (0.959 ‐ 1.178)
Delirium	Protein kinase C‐binding protein NELL1	WM	22	0.245	1.053 (0.965 ‐ 1.149)
Delirium	Protein kinase C‐binding protein NELL1	Weighted mode	22	0.255	1.056 (0.964 ‐ 1.157)

**Abbrevaitions**: Glial cell‐derived neurotrophic factor (GDNF); ciliary neurotrophic factor (CNTF); brain‐derived neurotrophic factor (BDNF); nerve growth factor (NGF); neurotrophin‐3 (NT‐3); neurotrophin‐4 (NT‐4); brain‐derived neurotrophic factor (BDNF); nerve growth factor (NGF); neural epidermal growth factor‐like 1 protein (NELL1); SNPs: single nucleotide polymorphisms; inverse‐variance weighted (IVW); weighted median (WM); leave‐one‐out (LOO);.

**TABLE 2:** Heterogeneity and pleiotropy analysis.

Exposure	Heterogeneity		Pleiotropy	
	Q	p	egger_intercept	p
NGF	5.776	0.672	0.039	0.199
BDNF	29.831	0.371	0.014	0.409
GDNF	19.782	0.286	−0.048	0.023
NT‐4	15.575	0.076	−0.045	0.458
Serum levels of protein NELL1	3.558	0.615	−0.006	0.780
NT‐3	16.539	0.621	0.006	0.740
CNTF	17.319	0.793	−0.005	0.845
NDNF	15.342	0.167	0.029	0.564

**Abbrevaitions**: Glial cell‐derived neurotrophic factor (GDNF); ciliary neurotrophic factor (CNTF); brain‐derived neurotrophic factor (BDNF); nerve growth factor (NGF); neurotrophin‐3 (NT‐3); neurotrophin‐4 (NT‐4); brain‐derived neurotrophic factor (BDNF); nerve growth factor (NGF); neural epidermal growth factor‐like 1 protein (NELL1).

Furthermore, LOO sensitivity analysis demonstrated that the results were robust and that no IVs significantly influenced the results (Figure S1–S9). In addition, MR‐PRESSO (Table 3) detected no outliers, further confirming the robustness of this data.

**TABLE 3:** MR‐PRESSO analysis.

Exposure	Raw		Outlier corrected		Global P	Number of outliers	Distortion P
	OR (CI%)	*P*	OR (CI%)	*P*			
BDNF	1 (0.91 ‐ 1.1)	0.971	/	/	0.375	/	/
CNTF	1 (0.94 ‐ 1.06)	0.962	/	/	0.870	/	/
GDNF	0.95 (0.81 ‐ 1.11)	0.526	/	/	0.325	/	/
NDNF	1.06 (0.87 ‐ 1.27)	0.583	/	/	0.223	/	/
NGF	0.89 (0.78 ‐ 1.03)	0.160	/	/	0.688	/	/
NT‐3	0.88 (0.75 ‐ 1.04)	0.148	/	/	0.637	/	/
NT‐4	0.99 (0.85 ‐ 1.14)	0.872	/	/	0.250	/	/
Protein kinase C‐binding protein NELL1	1.02 (0.97 ‐ 1.07)	0.392	/	/	0.926	/	/
Serum levels of protein NELL1	1.07 (0.98 ‐ 1.17)	0.215	/	/	0.711	/	/

**Abbrevaitions**: Glial cell‐derived neurotrophic factor (GDNF); ciliary neurotrophic factor (CNTF); brain‐derived neurotrophic factor (BDNF); nerve growth factor (NGF); neurotrophin‐3 (NT‐3); neurotrophin‐4 (NT‐4); brain‐derived neurotrophic factor (BDNF); nerve growth factor (NGF); neural epidermal growth factor‐like 1 protein (NELL1).

## 4 Discussion

The present study used the MR method to confirm a causal association between NTFs and delirium, but no significant association was found. Specifically, there was no significant association between protein kinase C‐binding protein NELL1, NT‐3, NT‐4 levels, and serum levels of NELL1, BDNF, NGF, CNTF, and GDNF, and delirium, while certain pleiotropy was observed for GDNF.

Neurotrophic factors promote brain plasticity by supporting the growth and connection of neurons. They are essential for maintaining cognitive function and memory. In patients with delirium, cognitive disturbances might be linked to impaired neuroplasticity, where neurotrophic factors could be involved in either protecting or failing to protect the brain during acute illness or injury (Xiao et al. 2023). BDNF is the most extensively studied NTF in relation to delirium. BDNF is a 13.5‐kDa member of the neurotrophin protein family abundantly present in the central nervous system (CNS), which has been reported to influence neuroplasticity and neurotransmission and to have a pivotal role in learning, memory, and cognition (Bathina and Das 2015). Decreased BDNF levels have been associated with increased oxidative stress, which has been implicated in the development of delirium (Pang et al. 2022). Furthermore, reductions in serum levels of BDNF have been associated with advanced age and decreases in the volume of the hippocampus and memory performance, all of which may predispose older individuals to develop delirium (Erickson et al. 2010). In fact, an association was found between intraoperative decline in BDNF and postoperative delirium, suggesting BDNF as a potential biomarker for delirium (Wyrobek et al. 2017). A different study (Williams et al. 2017) found no direct link between delirium and BDNF levels; however, recovery was less likely in those with continuously lower levels. According to the authors of that study, BDNF could be potentially used as a recovery marker, i.e., recovery from delirium could be predicted based on biological variables, thus having important clinical implications for the prognosis and treatment of delirium. Yet, even though previous studies have produced mixed results and reported inconclusive findings, they have highlighted the need for more rigorous research to disentangle complex relationships. To provide a genetic‐based assessment that minimizes confounding factors typically encountered in observational studies, we used the MR approach, which addresses confounding and reverse causality issues, thus providing more robust evidence for a potential protective effect of BDNF on delirium.

NGF is a pleiotropic neurotrophic protein that promotes the development, maintenance of function, and regeneration of nerve cells (Jockers‐Scherübl et al. 2007). Its relationship with delirium is still being explored, but there are several potential links that help explain how changes in NGF levels could influence the onset and progression of delirium. Since NGF is involved in the maintenance and function of cholinergic neurons, a reduction in NGF may worsen cholinergic dysfunction; this disruption could manifest as cognitive and behavioral symptoms, which are commonly seen in delirium, such as disorientation, inattention, and memory problems (Counts and Mufson 2005). It is also particularly interesting because its dual function affects neuron physiology and influences immune cell activity (Minnone et al. 2017). Numerous human and animal studies on inflammatory diseases have reported a significant increase in NGF synthesis in inflamed tissues (Fauchais et al. 2009). E.g., increased serum levels have been associated with CNS injuries such as stroke and epileptic seizures, as well as acute anxiety and acute stress response (Aloe et al. 1994). NGF can protect axons and myelin from inflammatory damage and modulate the immune system, thus decreasing the enhanced excitotoxicity during acute inflammatory activation (Terracina et al. 2023). Thus, it is believed that reduced NGF levels, whether due to inflammation, aging, or other factors, could contribute to delirium by impairing cognitive function and increasing the brain's vulnerability to stress and inflammation. However, NGF may act as a mediator in a more complex relationship (Minnone et al. 2017), which would also explain the lack of a significant causal relationship in the present study. This might also be true for NT‐4, for which we failed to find a significant causative effect on delirium in the present study. Nonetheless, a previous study on patients with schizophrenia found that the levels of NT‐4 and NGFβ in most patients were positively associated with levels of IL‐6, a well‐known proinflammatory cytokine (Malashenkova et al. 2019).

GDNF is another neurotrophic neuropeptide that has been implicated in the survival and differentiation of dopamine neurons in adults (Heberlein et al. 2010). Previous studies reported decreased serum levels of GDNF in subjects with mild cognitive impairment and Alzheimer's disease (Forlenza et al. 2015). In fact, it has been found that pathological conditions can substantially influence the level of GDNF in the CNS (Gurpreet Singh et al. 2023). In the present study, a certain level of pleiotropy was observed for GDNF, which is not surprising as GDNF has highly complex signaling and can bind to many different receptors, thus being able to induce pleiotropic effects (Anja Drinkut et al. 2016).

This MR study did not find a significant causal association between neurotrophic factors and delirium. The strengths of this study include the application of rigorous MR methodologies and the utilization of multiple sensitivity analyses to ensure robustness. This study also has limitations, such as insufficient power due to the limited number of IVs and the threshold for SNP selection used for certain factors. Yet, after filtering, we were able to identify only a limited number of SNPs in GWAS. Also, knowing that inflammation, a key player in delirium development, can impact neurotrophic factor levels (Xiao et al. 2023), and knowing that inflammatory cytokines (e.g., interleukin‐6, tumor necrosis factor‐alpha) may decrease the expression of neurotrophic factors like BDNF, understanding the potential mediator role of inflammation in the studied associations should be further explored. In addition, this study could have benefitted from complementary observational analyses or validation using alternative genetic methodologies. Thus, GWAS data from larger sample sizes and diverse populations must be obtained, and the results in clinical cohort studies must be validated.

Although this study did not yield significant findings, the results contribute to the growing body of knowledge on the genetic basis of delirium. Future studies could consider these factors and use our findings to investigate biomarkers or potential intervention targets. For example, while no direct causal relationships were observed, specific genetic variants or pathways identified in our study may warrant further exploration in larger cohorts or the context of different phenotypic subgroups. In addition, the lack of significant findings in this study does not diminish the value of the investigation but rather highlights the need for continued research. It is important to consider how these findings can shape future research priorities, especially in identifying biomarkers or intervention targets for delirium. Our study emphasizes the need for larger, more diverse populations and the integration of multi‐omics approaches to better understand the underlying genetic mechanisms. Additionally, targeted interventions based on these findings could help stratify patients for future personalized treatment strategies. Given the complexity of delirium and its multifactorial nature, future studies could focus on refining our approach by incorporating more precise genetic models or expanding the range of exposures and outcomes considered. Additionally, integrating functional genomics or longitudinal data could provide deeper insights into the temporal relationship between genetic factors and disease progression. These advancements could lead to identifying novel biomarkers or therapeutic targets, thereby increasing the clinical relevance of this research. In addition to DNA methylation modifications, histone modifications (such as methylation, acetylation, and phosphorylation) and ubiquitination modifications are also important epigenetic mechanisms that could play a critical role in the development and progression of diseases. Therefore, future research may need to focus more on the role of these epigenetic modifications.

## 5 Conclusion

No association between neurotrophic factors and delirium was identified. Yet, further research is needed to determine if these results apply to other races. Future research could benefit from more diverse ethnic samples, functional validation of implicated genetic variants, and exploration of potential mediating pathways. Subsequent studies and clinical trials are warranted further to elucidate the relationship between these exposure factors and delirium.

## Author Contributions

**Han Wu**: writing–original draft, conceptualization, writing–review and editing, methodology, software. **Ruilai Jiang**: conceptualization, writing–review and editing, writing–original draft. **Xiaocheng Huang**: conceptualization, writing–original draft, writing–review and editing, methodology. **Xiaogang Hu**: writing–original draft, writing–review and editing.

### Peer Review

The peer review history for this article is available at https://publons.com/publon/10.1002/brb3.70494

## Supporting information




**Figure S1**: The causal effect of BDNF levels on delirium. (A) Forest plot; (B) funnel plot; (C) LOO plot; and (D) scatter plot.

**Figure S2**: The causal effect of CNTF levels on delirium. (A) Forest plot; (B) funnel plot; (C) LOO plot; and (D) scatter plot.

**Figure S3**: The causal effect of GDNF levels on delirium. (A) Forest plot; (B) funnel plot; (C) LOO plot; and (D) scatter plot.

**Figure S4**: The causal effect of NDNF levels on delirium. (A) Forest plot; (B) funnel plot; (C) LOO plot; and (D) scatter plot.

**Figure S5**: The causal effect of NGF serum levels on delirium. (A) Forest plot; (B) funnel plot; (C) LOO plot; and (D) scatter plot.

**Figure S6**: The causal effect of NT‐3 serum levels on delirium. (A) Forest plot; (B) funnel plot; (C) LOO plot; and (D) scatter plot.

**Figure S7**: The causal effect of NT‐4 serum levels on delirium. (A) Forest plot; (B) funnel plot; (C) LOO plot; and (D) scatter plot.

**Figure S8**: The causal effect of protein kinase C‐binding protein NELL1 levels on delirium. (A) Forest plot; (B) funnel plot; (C) LOO plot; and (D) scatter plot.

**Figure S9**: The causal effect of serum levels of protein NELL1 on delirium. (A) Forest plot; (B) funnel plot; (C) LOO plot; and (D) scatter plot.

**Table S1**. Data source for exposure factors and outcomes.
**Table S2**. IV screening and F values.

## Data Availability

All data generated or analyzed during this study are included in this published article
