# Supplementary figures and images for: The Causal Relationship Between Neurotrophic Factors and Delirium: A Mendelian Randomization Study
Source: Brain Behav. 2025 May 5;15(5):e70494. doi: 10.1002/brb3.70494 (PMC12050958; doi:10.1002/brb3.70494)

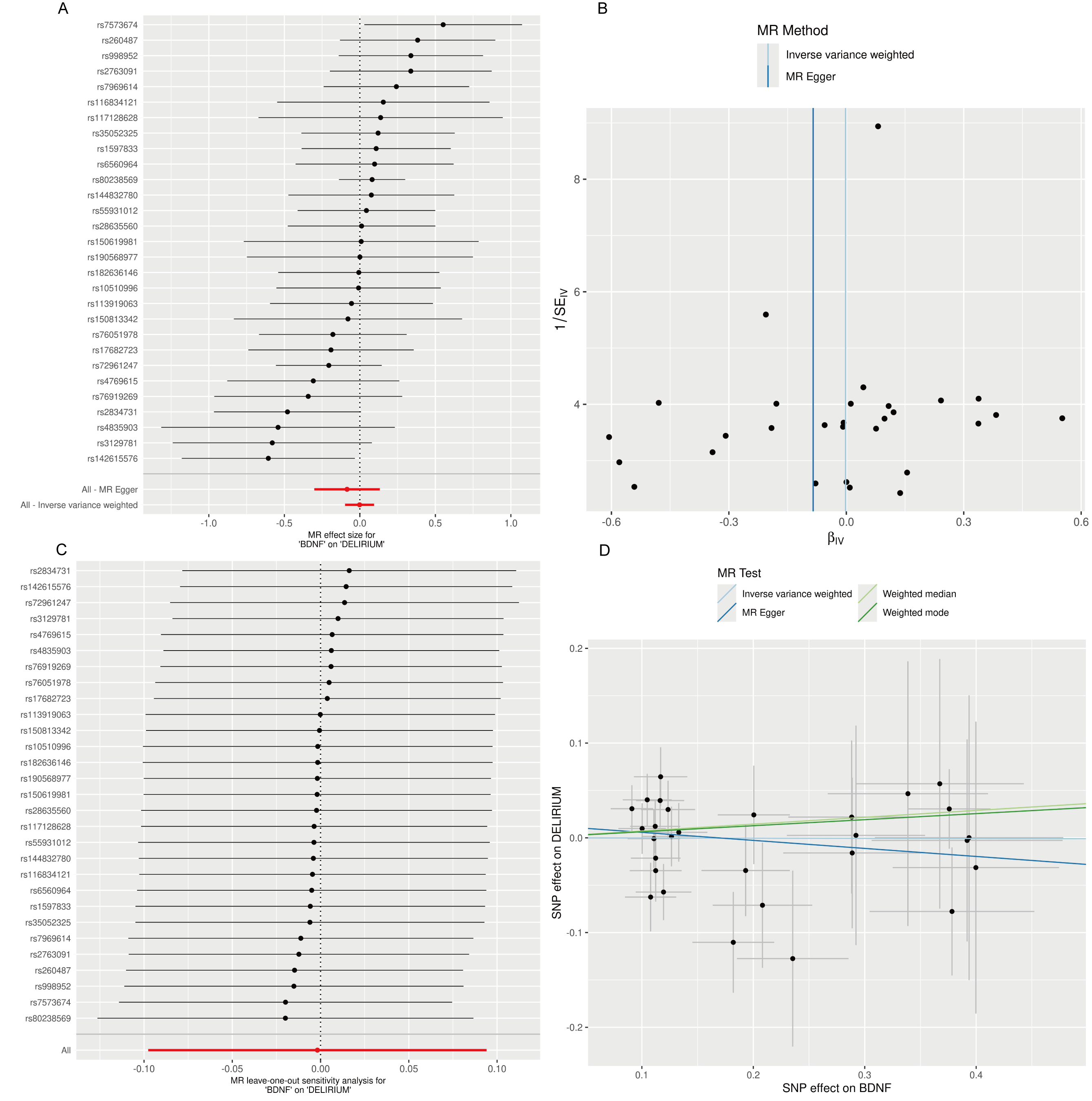

Supplement: Supplementary file 1 — Figure S1: The causal effect of BDNF levels on delirium. (A) Forest plot; (B) funnel plot; (C) LOO plot; and (D) scatter plot. [file BRB3-15-e70494-s010.tif]

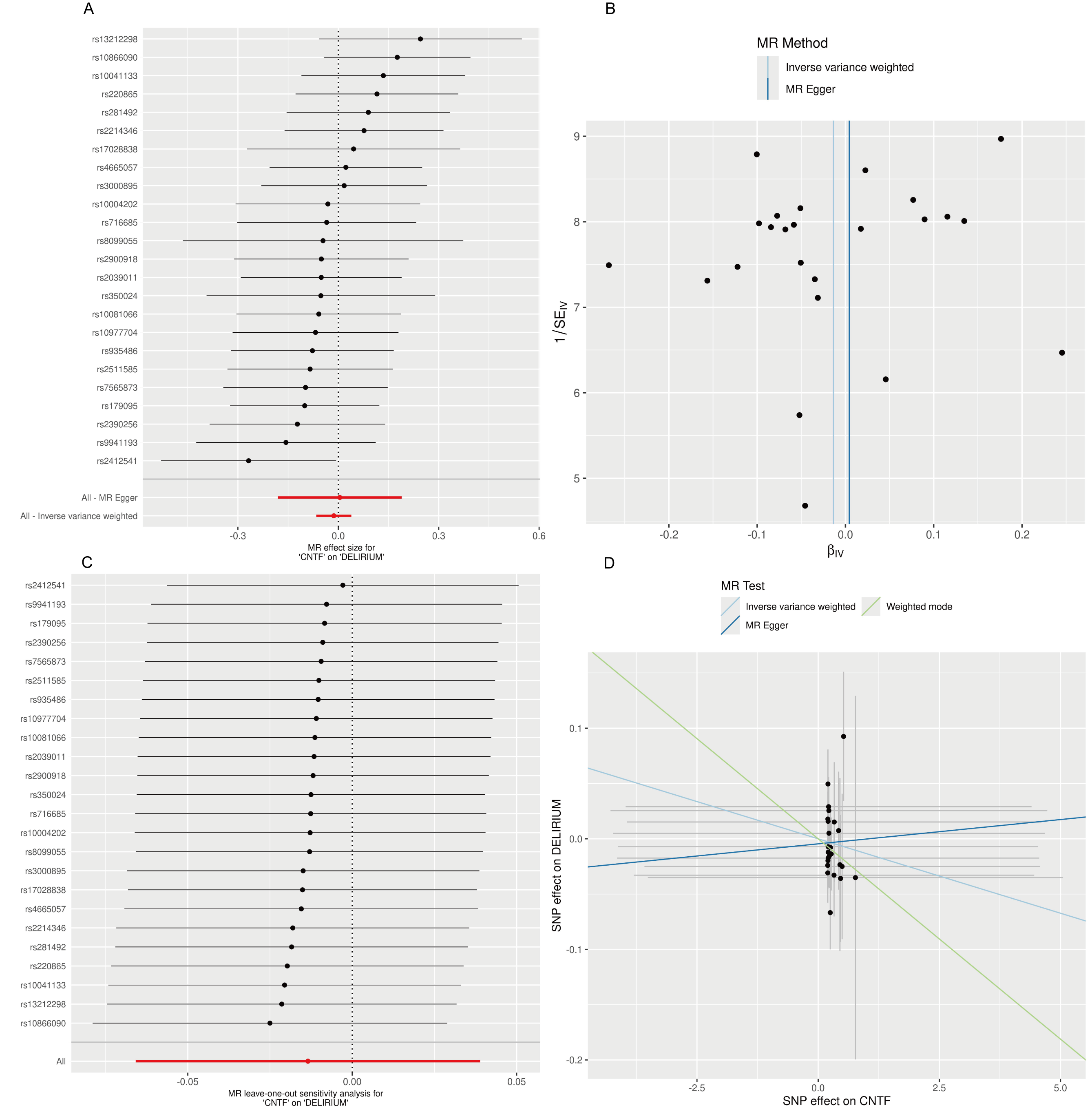

Supplement: Supplementary file 2 — Figure S2: The causal effect of CNTF levels on delirium. (A) Forest plot; (B) funnel plot; (C) LOO plot; and (D) scatter plot. [file BRB3-15-e70494-s006.tif]

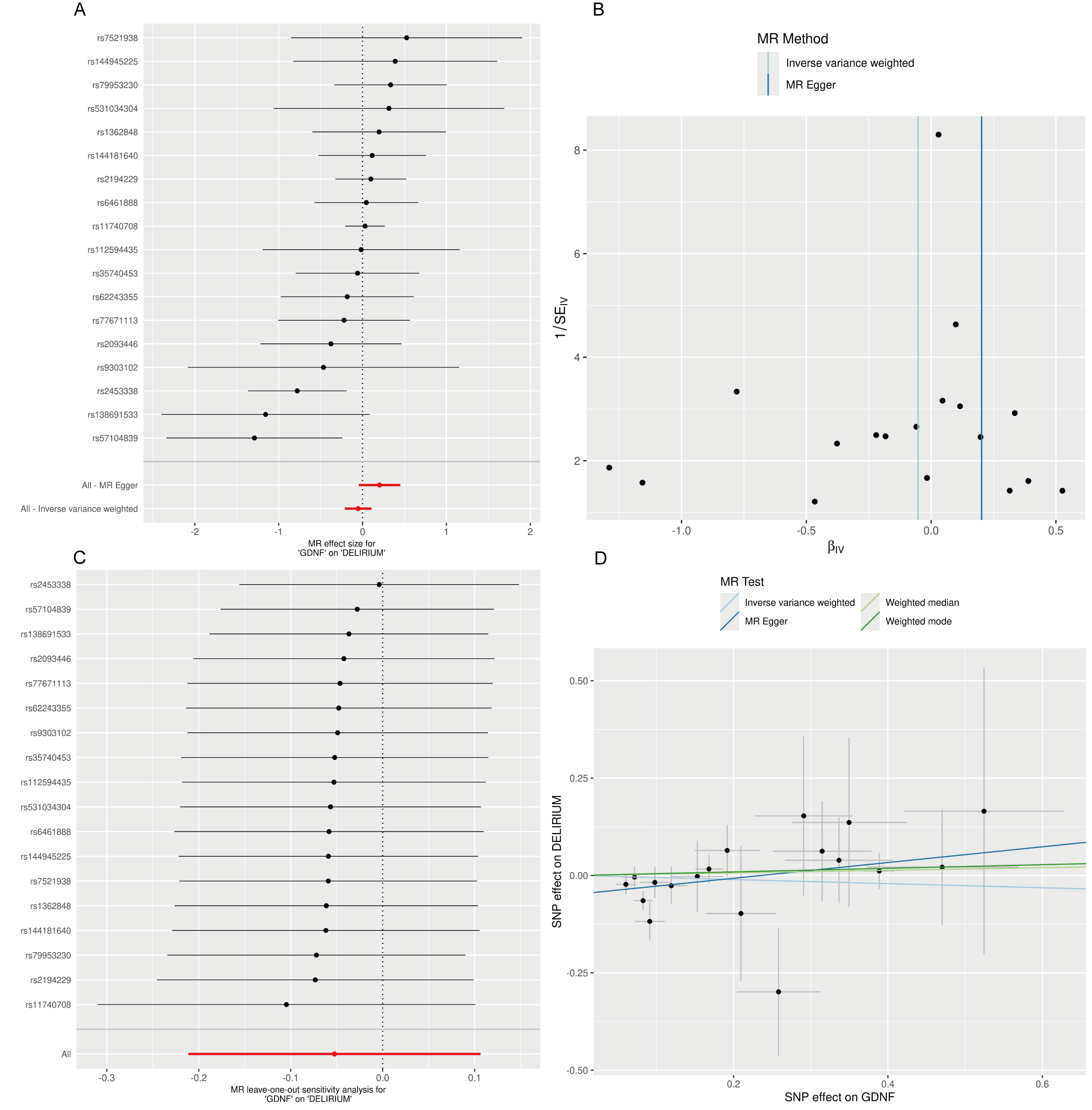

Supplement: Supplementary file 3 — Figure S3: The causal effect of GDNF levels on delirium. (A) Forest plot; (B) funnel plot; (C) LOO plot; and (D) scatter plot. [file BRB3-15-e70494-s009.tif]

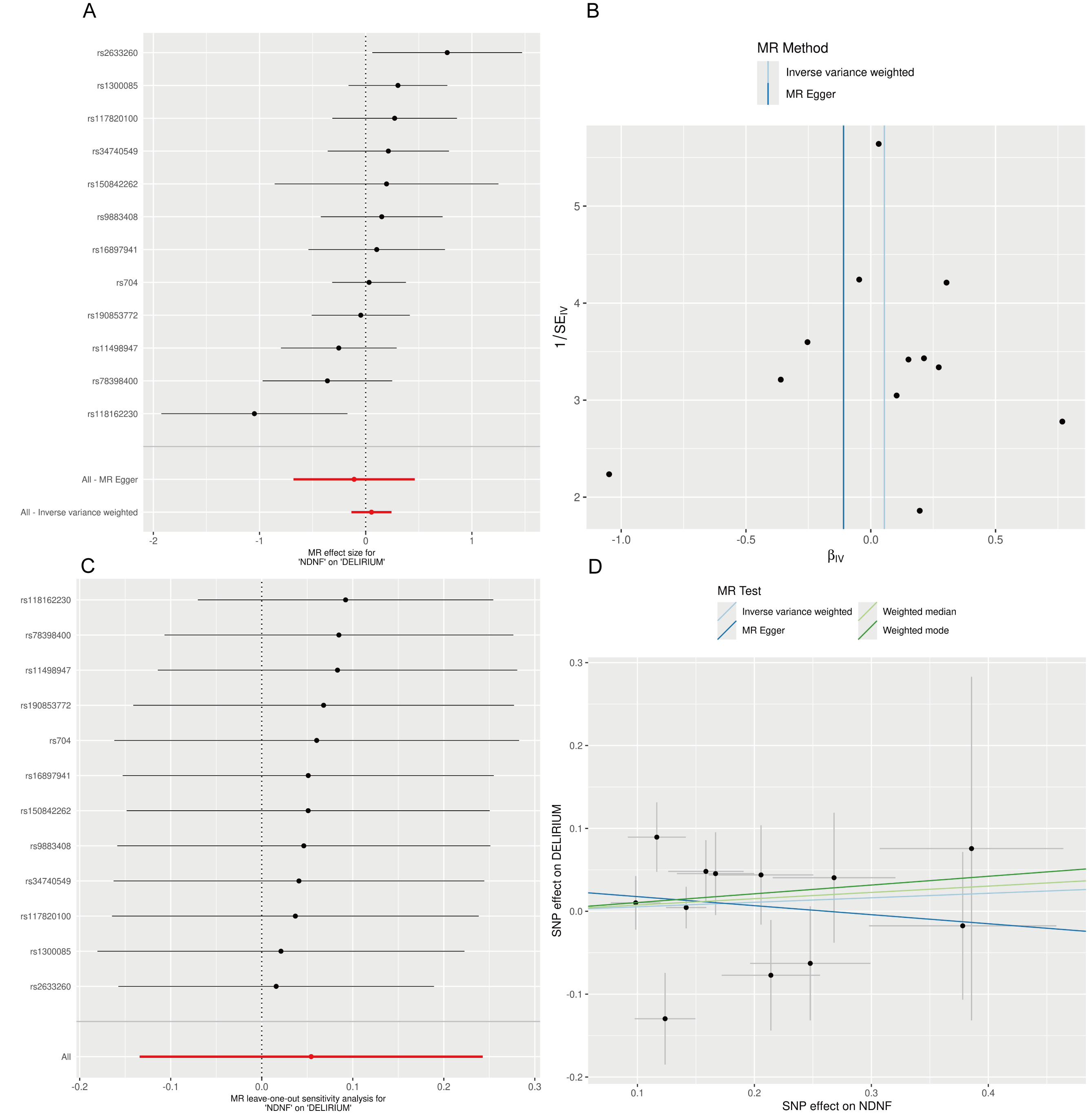

Supplement: Supplementary file 4 — Figure S4: The causal effect of NDNF levels on delirium. (A) Forest plot; (B) funnel plot; (C) LOO plot; and (D) scatter plot. [file BRB3-15-e70494-s005.tif]

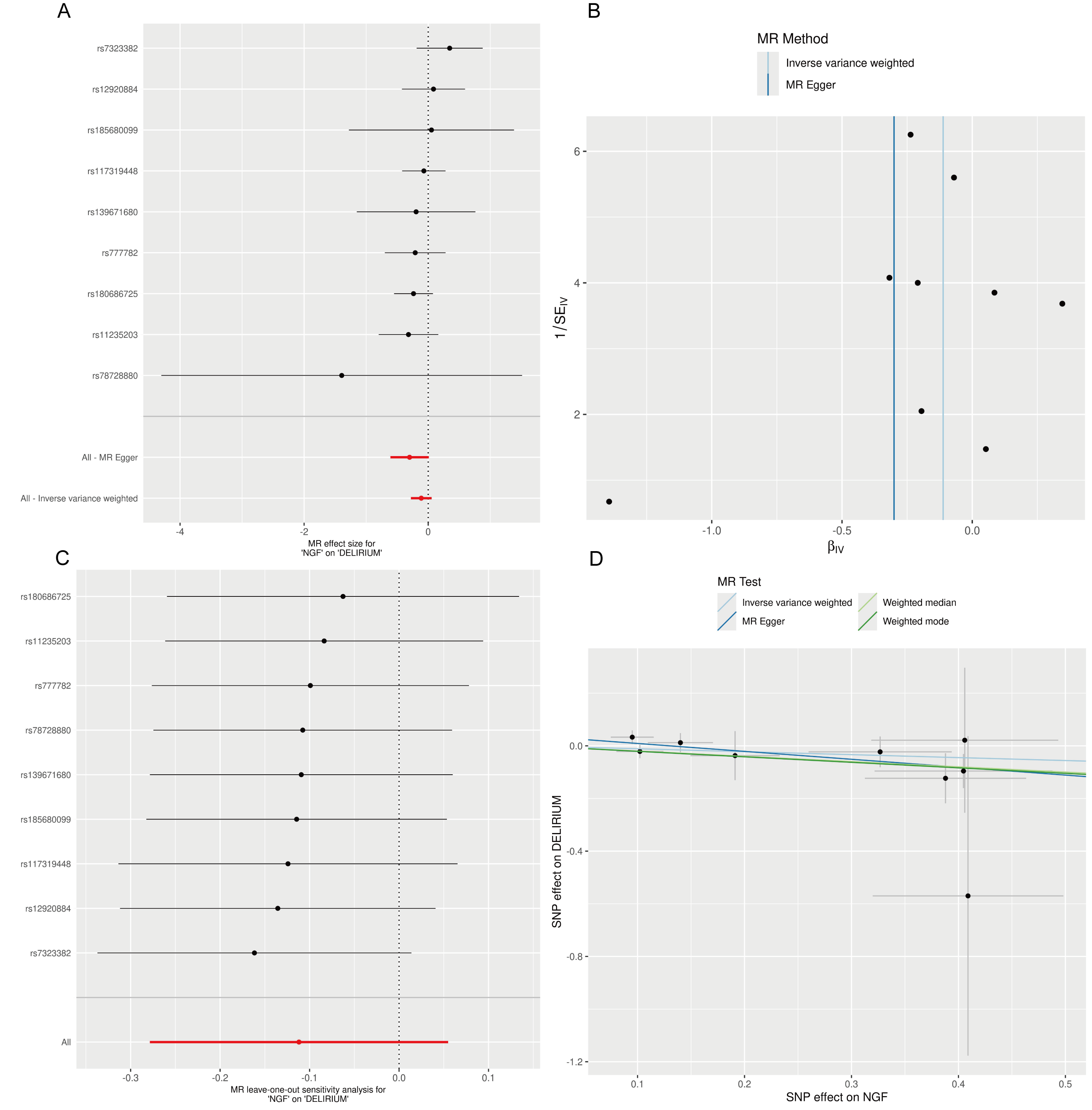

Supplement: Supplementary file 5 — Figure S5: The causal effect of NGF serum levels on delirium. (A) Forest plot; (B) funnel plot; (C) LOO plot; and (D) scatter plot. [file BRB3-15-e70494-s008.tif]

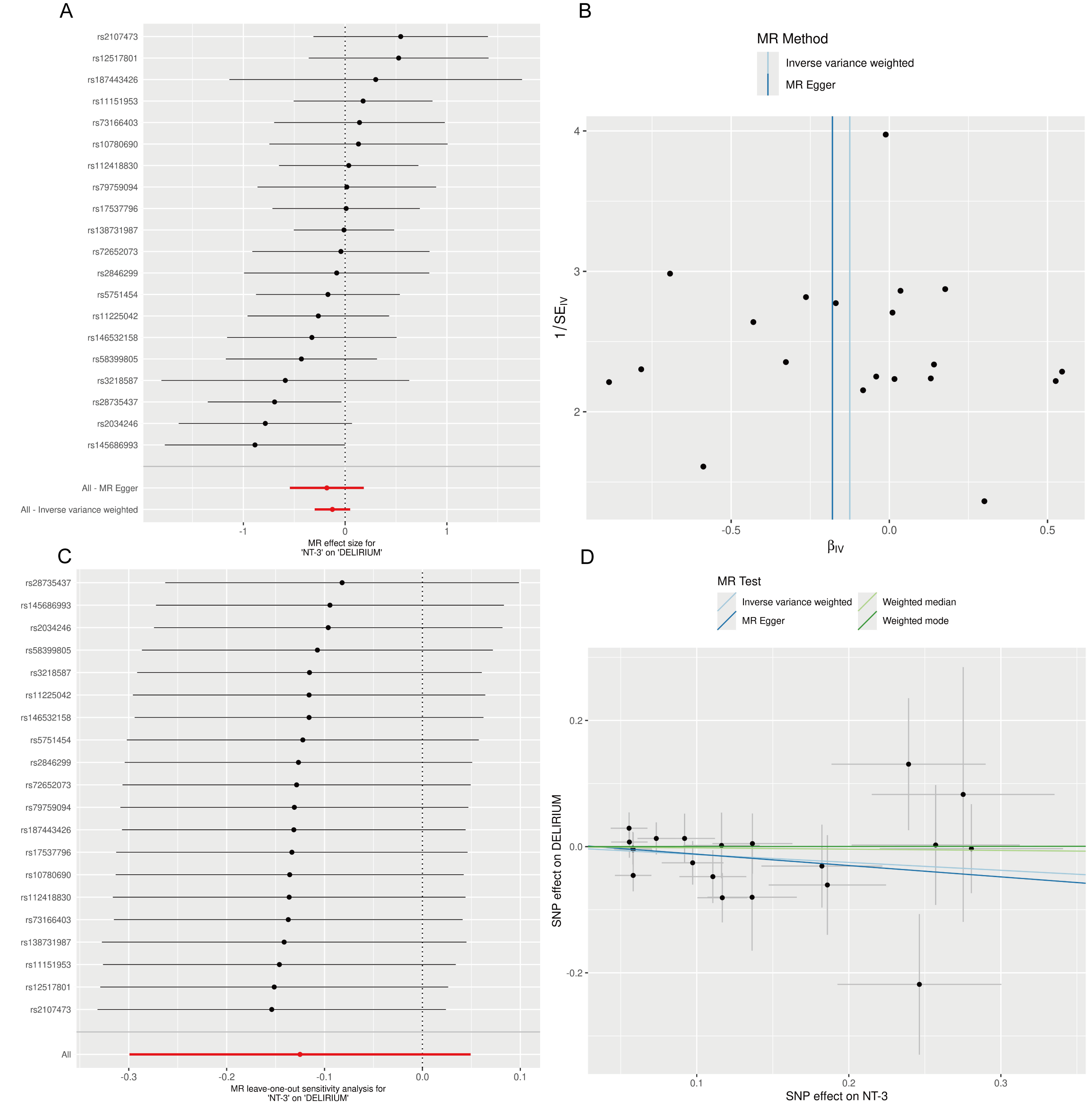

Supplement: Supplementary file 6 — Figure S6: The causal effect of NT‐3 serum levels on delirium. (A) Forest plot; (B) funnel plot; (C) LOO plot; and (D) scatter plot. [file BRB3-15-e70494-s002.tif]

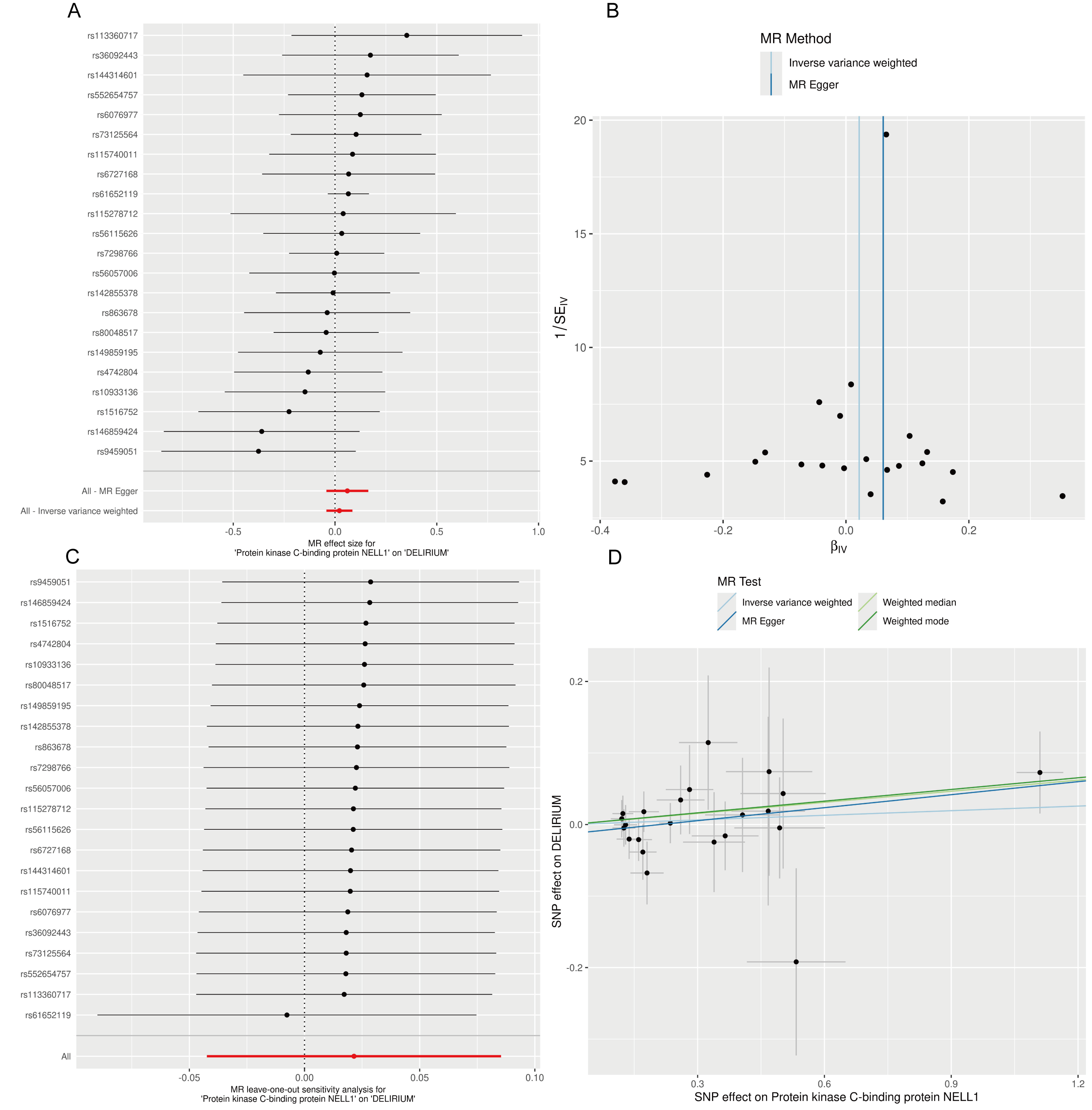

Supplement: Supplementary file 7 — Figure S7: The causal effect of NT‐4 serum levels on delirium. (A) Forest plot; (B) funnel plot; (C) LOO plot; and (D) scatter plot. [file BRB3-15-e70494-s004.tif]

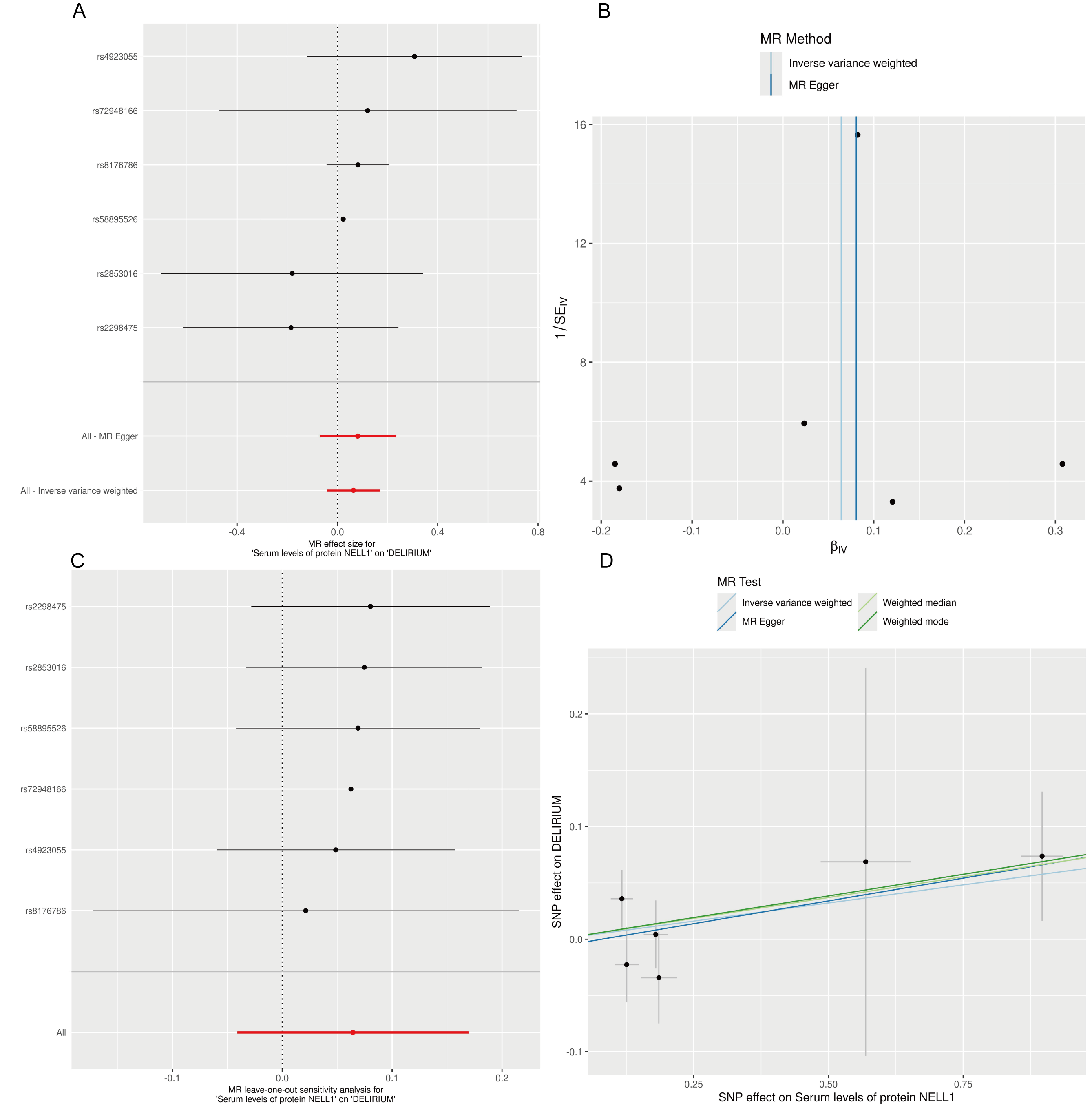

Supplement: Supplementary file 8 — Figure S8: The causal effect of protein kinase C‐binding protein NELL1 levels on delirium. (A) Forest plot; (B) funnel plot; (C) LOO plot; and (D) scatter plot. [file BRB3-15-e70494-s001.tif]

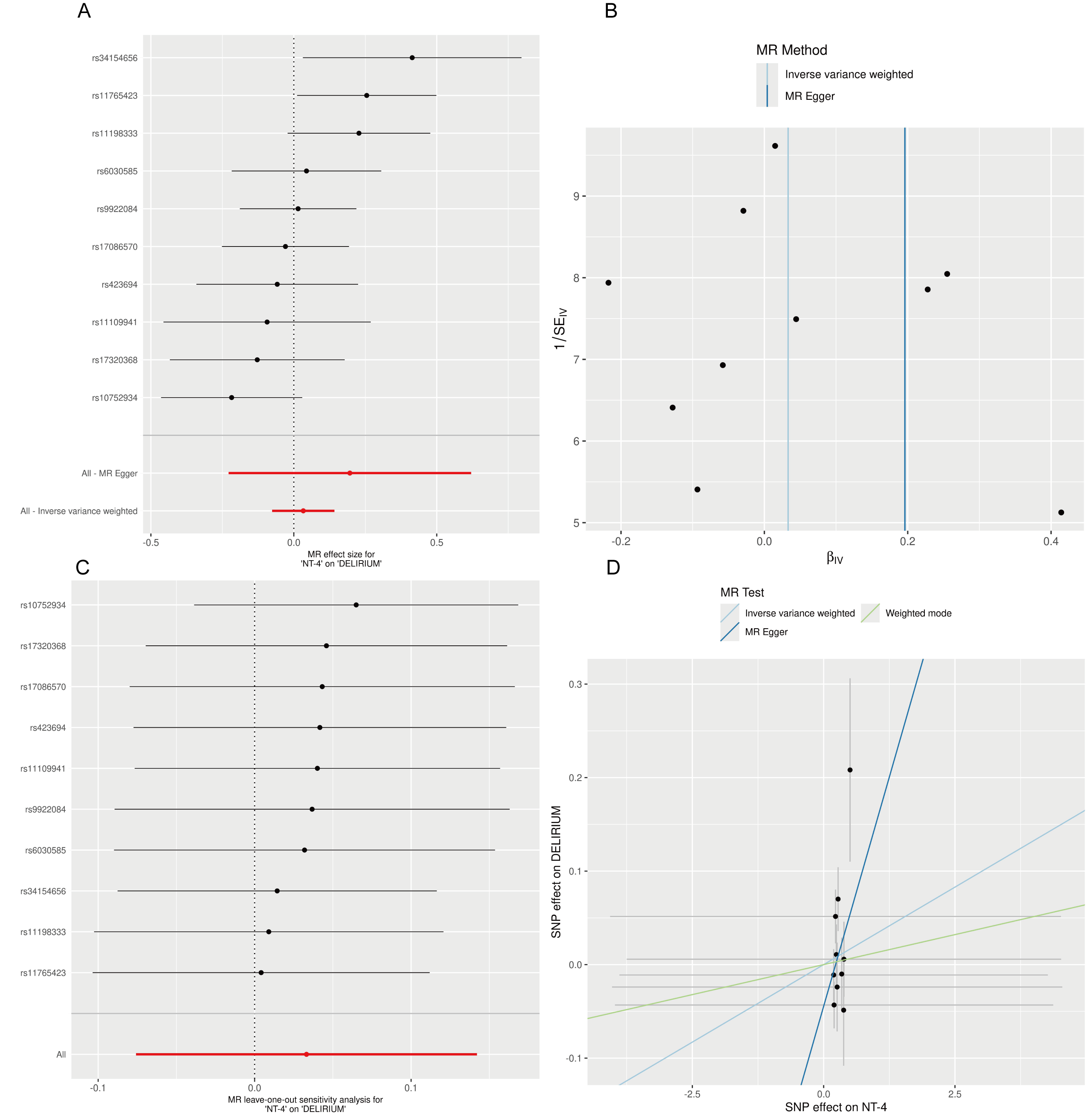

Supplement: Supplementary file 9 — Figure S9: The causal effect of serum levels of protein NELL1 on delirium. (A) Forest plot; (B) funnel plot; (C) LOO plot; and (D) scatter plot. [file BRB3-15-e70494-s007.tif]
