# Supplementary material for: The Causal Relationship Between Neurotrophic Factors and Delirium: A Mendelian Randomization Study
Source: Brain Behav. 2025 May 5;15(5):e70494. doi: 10.1002/brb3.70494 (PMC12050958; doi:10.1002/brb3.70494)
Supplement: Supplementary file 10 — Table S1. Data source for exposure factors and outcomes. Table S2. IV screening and F values. [file BRB3-15-e70494-s003.docx]

**Table S1.** Data source

| **Trait** | **GWAS ID** | **Sample size case/control）** | **SNPs number** | **PMID** |
| --- | --- | --- | --- | --- |
| Delirium | F5_DELIRIUM | 3371/388560 | NA | NA |
| Serum levels of protein NELL1 | GCST90089486 | 5364 | NA | 35078996 |
| Protein kinase C-binding protein NELL1 | GCST90242481 | 3301 | NA | 29875488 |
| Serum levels of BDNF | GCST90087740 | 5366 | NA | 35078996 |
| Serum levels of NGF | GCST90089211 | 5365 | NA | 35078996 |
| Serum levels of NDNF | GCST90089524 | 5353 | NA | 35078996 |
| CNTF levels | GCST90100830 | 997 | NA | 28240269 |
| GDNF levels | GCST90274792 | 14736 | NA | 37563310 |
| NT-3 levels | GCST90274829 | 33987 | NA | 37563310 |
| NT-4 levels | GCST90101363 | 997 | NA | 28240269 |

**Abbrevaitions:** Glial cell-derived neurotrophic factor (GDNF); ciliary neurotrophic factor (CNTF); brain-derived neurotrophic factor (BDNF); nerve growth factor (NGF); neurotrophin-3 (NT-3); neurotrophin-4 (NT-4); brain-derived neurotrophic factor (BDNF); nerve growth factor (NGF); neural epidermal growth factor-like 1 protein (NELL1)

**Table S2.** IV screening and F values

| **Exposure factor** | **Outcome** | **Initially selected SNP** | **mean_F** | **min_F** | **max_F** | **The number of proxy SNPs that did not matched** | **Finally selected SNP** |
| --- | --- | --- | --- | --- | --- | --- | --- |
| Serum levels of protein NELL1 | Delirium | 6 | 120.97 | 30.51 | 521.74 | 0 | 6 |
| Protein kinase C-binding protein NELL1 | Delirium | 22 | 42.57 | 20.86 | 402.51 | 0 | 22 |
| Serum levels of BDNF | Delirium | 31 | 26.70 | 20.92 | 103.16 | 2 | 29 |
| Serum levels of NGF | Delirium | 9 | 22.52 | 20.94 | 26.48 | 0 | 9 |
| Serum levels of NDNF | Delirium | 12 | 27.17 | 21.13 | 67.67 | 0 | 12 |
| CNTF levels | Delirium | 24 | 18.51 | 16.46 | 22.62 | 0 | 24 |
| GDNF levels | Delirium | 22 | 44.22 | 20.85 | 385.53 | 4 | 18 |
| NT-3 levels | Delirium | 22 | 24.22 | 20.90 | 50.72 | 2 | 20 |
| NT-4 levels | Delirium | 10 | 18.05 | 16.46 | 22.76 | 0 | 10 |

**Abbrevaitions:** Glial cell-derived neurotrophic factor (GDNF); ciliary neurotrophic factor (CNTF); brain-derived neurotrophic factor (BDNF); nerve growth factor (NGF); neurotrophin-3 (NT-3); neurotrophin-4 (NT-4); brain-derived neurotrophic factor (BDNF); nerve growth factor (NGF); neural epidermal growth factor-like 1 protein (NELL1)

**Table S3.** IV screening (more details)

| SNP | pval.exposure | chr.exposure | pos.exposure | effect_allele.exposure | other_allele.exposure | eaf.exposure | beta.exposure | se.exposure | exposure | mr_keep.exposure | pval_origin.exposure | id.exposure | samplesize.exposure | r2 | F |
| --- | --- | --- | --- | --- | --- | --- | --- | --- | --- | --- | --- | --- | --- | --- | --- |
| rs2853016 | 1,64904E-08 | 11 | 20729686 | C | T | 0,2205 | 0,125561 | 0,0222076 | Serum levels of protein NELL1 | TRUE | reported | mhQG5i | 5364 | 0,005924299 | 31,95540451 |
| rs4923055 | 2,11787E-08 | 11 | 20822918 | A | G | 0,2665 | -0,116737 | 0,0208066 | Serum levels of protein NELL1 | TRUE | reported | mhQG5i | 5364 | 0,005834248 | 31,46682036 |
| rs8176786 | 2,6002E-110 | 11 | 20959394 | T | C | 0,05454 | 0,896424 | 0,0392377 | Serum levels of protein NELL1 | TRUE | reported | mhQG5i | 5364 | 0,088675592 | 521,7445323 |
| rs72948166 | 1,15107E-11 | 11 | 21094538 | G | T | 0,01273 | -0,569132 | 0,0836814 | Serum levels of protein NELL1 | TRUE | reported | mhQG5i | 5364 | 0,008549689 | 46,23875964 |
| rs2298475 | 3,46473E-08 | 11 | 126278203 | C | T | 0,08151 | -0,185321 | 0,0335467 | Serum levels of protein NELL1 | TRUE | reported | mhQG5i | 5364 | 0,005657142 | 30,50617102 |
| rs58895526 | 1,57326E-15 | 12 | 658104 | G | A | 0,2168 | 0,179608 | 0,0224646 | Serum levels of protein NELL1 | TRUE | reported | mhQG5i | 5364 | 0,011776611 | 63,89869874 |
| rs115278712 | 8,31764E-08 | 1 | 70561800 | A | G | 0,015900001 | -0,467 | 0,0871 | Protein kinase C-binding protein NELL1 | TRUE | reported | QSGEN4 | 3301 | 0,008633486 | 28,72990847 |
| rs113360717 | 2,5704E-06 | 2 | 25834301 | T | C | 0,028829999 | -0,325 | 0,0691 | Protein kinase C-binding protein NELL1 | TRUE | reported | QSGEN4 | 3301 | 0,006656784 | 22,10789628 |
| rs863678 | 3,46737E-06 | 2 | 176109376 | T | G | 0,6153 | -0,1255 | 0,0271 | Protein kinase C-binding protein NELL1 | TRUE | reported | QSGEN4 | 3301 | 0,006454921 | 21,43313311 |
| rs6727168 | 3,89045E-06 | 2 | 207124746 | T | C | 0,319099993 | 0,1205 | 0,0261 | Protein kinase C-binding protein NELL1 | TRUE | reported | QSGEN4 | 3301 | 0,006415821 | 21,30246549 |
| rs10933136 | 3,46737E-06 | 2 | 226319372 | C | G | 0,247500002 | 0,138 | 0,0297 | Protein kinase C-binding protein NELL1 | TRUE | reported | QSGEN4 | 3301 | 0,006497833 | 21,57655305 |
| rs552654757 | 4,36516E-06 | 3 | 5119217 | A | G | 0,079520002 | 0,2598 | 0,0566 | Protein kinase C-binding protein NELL1 | TRUE | reported | QSGEN4 | 3301 | 0,006342154 | 21,05630789 |
| rs73125564 | 1,28825E-06 | 4 | 32591008 | A | T | 0,158999994 | 0,1728 | 0,0357 | Protein kinase C-binding protein NELL1 | TRUE | reported | QSGEN4 | 3301 | 0,007047482 | 23,41465889 |
| rs146859424 | 4,89779E-06 | 4 | 146616471 | T | G | 0,017890001 | -0,5332 | 0,1167 | Protein kinase C-binding protein NELL1 | TRUE | reported | QSGEN4 | 3301 | 0,006284275 | 20,86293287 |
| rs142855378 | 4,0738E-06 | 5 | 102448336 | A | C | 0,017890001 | -0,494 | 0,1073 | Protein kinase C-binding protein NELL1 | TRUE | reported | QSGEN4 | 3301 | 0,006380125 | 21,18318432 |
| rs115740011 | 6,76083E-07 | 6 | 41739406 | A | G | 0,015900001 | 0,5023 | 0,1011 | Protein kinase C-binding protein NELL1 | TRUE | reported | QSGEN4 | 3301 | 0,007422377 | 24,66952778 |
| rs9459051 | 4,57088E-06 | 6 | 164267074 | T | C | 0,124300003 | 0,1803 | 0,0394 | Protein kinase C-binding protein NELL1 | TRUE | reported | QSGEN4 | 3301 | 0,006303868 | 20,9283891 |
| rs144314601 | 4,36516E-06 | 7 | 24262015 | A | G | 0,016899999 | 0,4691 | 0,1021 | Protein kinase C-binding protein NELL1 | TRUE | reported | QSGEN4 | 3301 | 0,006354267 | 21,09677995 |
| rs80048517 | 3,80189E-06 | 7 | 110494769 | T | C | 0,025839999 | -0,3652 | 0,079 | Protein kinase C-binding protein NELL1 | TRUE | reported | QSGEN4 | 3301 | 0,006432197 | 21,35719172 |
| rs4742804 | 4,36516E-07 | 9 | 101108037 | T | C | 0,1998 | -0,1604 | 0,0317 | Protein kinase C-binding protein NELL1 | TRUE | reported | QSGEN4 | 3301 | 0,007696428 | 25,58744927 |
| rs61652119 | 1,28825E-89 | 11 | 20933724 | A | G | 0,053679999 | 1,1098 | 0,0553 | Protein kinase C-binding protein NELL1 | TRUE | reported | QSGEN4 | 3301 | 0,108741951 | 402,5093467 |
| rs1516752 | 1,65959E-07 | 11 | 21063672 | C | G | 0,156100005 | 0,1704 | 0,0326 | Protein kinase C-binding protein NELL1 | TRUE | reported | QSGEN4 | 3301 | 0,008208782 | 27,30491142 |
| rs7298766 | 1,54882E-18 | 12 | 552490 | A | G | 0,328999996 | -0,2354 | 0,0268 | Protein kinase C-binding protein NELL1 | TRUE | reported | QSGEN4 | 3301 | 0,022838324 | 77,10457012 |
| rs149859195 | 3,71535E-06 | 14 | 81562371 | A | G | 0,02485 | 0,3389 | 0,0733 | Protein kinase C-binding protein NELL1 | TRUE | reported | QSGEN4 | 3301 | 0,006434078 | 21,36347907 |
| rs56115626 | 4,46684E-06 | 15 | 69500072 | T | C | 0,02187 | -0,4062 | 0,0885 | Protein kinase C-binding protein NELL1 | TRUE | reported | QSGEN4 | 3301 | 0,006341397 | 21,05378036 |
| rs36092443 | 6,16595E-07 | 17 | 49388236 | A | G | 0,042739999 | -0,2809 | 0,0563 | Protein kinase C-binding protein NELL1 | TRUE | reported | QSGEN4 | 3301 | 0,007484768 | 24,87845922 |
| rs6076977 | 6,0256E-07 | 20 | 704472 | A | G | 0,510900021 | 0,1231 | 0,0247 | Protein kinase C-binding protein NELL1 | TRUE | reported | QSGEN4 | 3301 | 0,007468288 | 24,82326997 |
| rs56057006 | 2,51189E-06 | 20 | 47081652 | A | G | 0,2465 | -0,1296 | 0,0275 | Protein kinase C-binding protein NELL1 | TRUE | reported | QSGEN4 | 3301 | 0,006683237 | 22,19634194 |
| rs113919063 | 3,19463E-06 | 2 | 68874107 | A | G | 0,02458 | -0,288761 | 0,0619283 | BDNF | TRUE | reported | aRJjZa | 5366 | 0,00403545 | 21,73385966 |
| rs7573674 | 1,06741E-06 | 2 | 160687525 | A | G | 0,2041 | 0,116826 | 0,0239179 | BDNF | TRUE | reported | aRJjZa | 5366 | 0,00442645 | 23,84904668 |
| rs190568977 | 3,32109E-06 | 3 | 4843485 | T | A | 0,01317 | -0,393634 | 0,0845651 | BDNF | TRUE | reported | aRJjZa | 5366 | 0,00402163 | 21,65912831 |
| rs35052325 | 6,27249E-10 | 3 | 39171676 | T | C | 0,09733 | -0,200475 | 0,0323621 | BDNF | TRUE | reported | aRJjZa | 5366 | 0,007100707 | 38,36057904 |
| rs10510996 | 2,71957E-06 | 3 | 70462667 | T | C | 0,2129 | 0,110798 | 0,0235943 | BDNF | TRUE | reported | aRJjZa | 5366 | 0,004092775 | 22,04386531 |
| rs76919269 | 3,28141E-06 | 3 | 104903457 | C | G | 0,04862 | -0,208226 | 0,0447096 | BDNF | TRUE | reported | aRJjZa | 5366 | 0,004025925 | 21,68235471 |
| rs150619981 | 2,56313E-06 | 4 | 176976478 | A | C | 0,02422 | 0,292147 | 0,0620516 | BDNF | TRUE | reported | aRJjZa | 5366 | 0,00411392 | 22,15822634 |
| rs28635560 | 2,46292E-06 | 4 | 183544419 | C | G | 0,1504 | -0,126585 | 0,0268399 | BDNF | TRUE | reported | aRJjZa | 5366 | 0,00412815 | 22,23518801 |
| rs4835903 | 2,61571E-06 | 5 | 125792609 | A | G | 0,03819 | 0,235385 | 0,0500397 | BDNF | TRUE | reported | aRJjZa | 5366 | 0,004106675 | 22,11903999 |
| rs2763091 | 1,72274E-06 | 6 | 7534146 | C | A | 0,4843 | -0,091051 | 0,0190132 | BDNF | TRUE | reported | aRJjZa | 5366 | 0,004255555 | 22,92435407 |
| rs3129781 | 2,74044E-06 | 6 | 32648500 | G | T | 0,2234 | 0,107862 | 0,0229767 | BDNF | TRUE | reported | aRJjZa | 5366 | 0,004090073 | 22,02925098 |
| rs117128628 | 2,56313E-06 | 6 | 143270401 | G | A | 0,01768 | -0,338755 | 0,0719512 | BDNF | TRUE | reported | aRJjZa | 5366 | 0,004113906 | 22,15814735 |
| rs182636146 | 4,87742E-06 | 8 | 22728934 | A | G | 0,01282 | 0,39188 | 0,0856632 | BDNF | TRUE | reported | aRJjZa | 5366 | 0,00388487 | 20,91971252 |
| rs1597833 | 7,01471E-07 | 8 | 53670728 | G | A | 0,2382 | 0,111981 | 0,0225454 | BDNF | TRUE | reported | aRJjZa | 5366 | 0,004576464 | 24,66101196 |
| rs116834121 | 1,15255E-06 | 8 | 125570293 | A | G | 0,01662 | -0,367344 | 0,075442 | BDNF | TRUE | reported | aRJjZa | 5366 | 0,004399002 | 23,70050587 |
| rs998952 | 7,40611E-08 | 9 | 101084801 | C | G | 0,2649 | -0,11639 | 0,0215995 | BDNF | TRUE | reported | aRJjZa | 5366 | 0,005382073 | 29,02565583 |
| rs150813342 | 9,00119E-08 | 9 | 135864513 | T | C | 0,01644 | -0,399745 | 0,0746739 | BDNF | TRUE | reported | aRJjZa | 5366 | 0,00531208 | 28,64616663 |
| rs55931012 | 2,21391E-07 | 9 | 136949669 | T | C | 0,1709 | -0,133127 | 0,0256647 | BDNF | TRUE | reported | aRJjZa | 5366 | 0,00498927 | 26,89663859 |
| rs76051978 | 1,14306E-06 | 10 | 57745956 | A | C | 0,06268 | 0,19313 | 0,03965 | BDNF | TRUE | reported | aRJjZa | 5366 | 0,004401964 | 23,71653281 |
| rs144832780 | 4,19672E-07 | 10 | 92700871 | T | C | 0,02855 | 0,288274 | 0,0569026 | BDNF | TRUE | reported | aRJjZa | 5366 | 0,004760185 | 25,65575668 |
| rs142615576 | 7,30096E-07 | 11 | 27708060 | C | T | 0,07205 | 0,182048 | 0,0367099 | BDNF | TRUE | reported | aRJjZa | 5366 | 0,004562143 | 24,58348912 |
| rs80238569 | 5,01072E-24 | 11 | 27777863 | G | A | 0,07134 | 0,375866 | 0,0370003 | BDNF | TRUE | reported | aRJjZa | 5366 | 0,018868277 | 103,1558102 |
| rs72961247 | 3,22634E-07 | 11 | 37536790 | G | A | 0,01688 | 0,378313 | 0,0739442 | BDNF | TRUE | reported | aRJjZa | 5366 | 0,004854341 | 26,16570267 |
| rs17682723 | 4,76848E-07 | 11 | 123706758 | G | T | 0,2412 | 0,112382 | 0,0222909 | BDNF | TRUE | reported | aRJjZa | 5366 | 0,004714496 | 25,40834672 |
| rs7969614 | 3,60089E-07 | 12 | 44838183 | G | A | 0,1918 | 0,123506 | 0,024239 | BDNF | TRUE | reported | aRJjZa | 5366 | 0,004815039 | 25,95283564 |
| rs4769615 | 1,46416E-06 | 13 | 29161880 | T | C | 0,2123 | 0,112414 | 0,0233152 | BDNF | TRUE | reported | aRJjZa | 5366 | 0,004313546 | 23,23809984 |
| rs6560964 | 2,58821E-06 | 13 | 41445268 | T | C | 0,2969 | -0,10025 | 0,0213021 | BDNF | TRUE | reported | aRJjZa | 5366 | 0,004110403 | 22,13920518 |
| rs77184962 | 3,40604E-06 | 16 | 80562446 | T | C | 0,05614 | 0,193974 | 0,0417187 | BDNF | TRUE | reported | aRJjZa | 5366 | 0,004012626 | 21,61044004 |
| rs260487 | 1,88439E-06 | 19 | 58685347 | G | A | 0,2529 | -0,104816 | 0,0219707 | BDNF | TRUE | reported | aRJjZa | 5366 | 0,004223559 | 22,75126123 |
| rs2834731 | 1,82247E-06 | 21 | 36383142 | C | T | 0,1849 | -0,119436 | 0,0249997 | BDNF | TRUE | reported | aRJjZa | 5366 | 0,004235521 | 22,81597366 |
| rs3002417 | 1,15038E-06 | X | 39708724 | C | T | 0,3943 | 0,0796202 | 0,0163505 | BDNF | TRUE | reported | aRJjZa | 5366 | 0,004399657 | 23,70404993 |
| rs139671680 | 4,83894E-06 | 5 | 66961825 | A | G | 0,05481 | 0,191162 | 0,0417719 | NGF | TRUE | reported | ABlBR7 | 5365 | 0,003888418 | 20,93498927 |
| rs185680099 | 3,53525E-06 | 9 | 101596108 | A | T | 0,0122 | 0,405885 | 0,0874402 | NGF | TRUE | reported | ABlBR7 | 5365 | 0,004000122 | 21,53881418 |
| rs117319448 | 1,0676E-06 | 10 | 49907670 | A | G | 0,01989 | 0,326786 | 0,0669039 | NGF | TRUE | reported | ABlBR7 | 5365 | 0,004427181 | 23,84855589 |
| rs11235203 | 2,74265E-07 | 11 | 87263143 | C | T | 0,01706 | 0,387909 | 0,0753673 | NGF | TRUE | reported | ABlBR7 | 5365 | 0,004913432 | 26,48084677 |
| rs777782 | 3,46481E-06 | 13 | 60843474 | A | T | 0,2519 | 0,102188 | 0,0219946 | NGF | TRUE | reported | ABlBR7 | 5365 | 0,004007321 | 21,57772893 |
| rs7323382 | 2,29742E-06 | 13 | 106477943 | T | C | 0,3385 | -0,0950445 | 0,0200919 | NGF | TRUE | reported | ABlBR7 | 5365 | 0,004153695 | 22,36917852 |
| rs12920884 | 4,24092E-06 | 16 | 17383052 | A | G | 0,1096 | 0,140074 | 0,0304247 | NGF | TRUE | reported | ABlBR7 | 5365 | 0,003935322 | 21,18851513 |
| rs78728880 | 4,58342E-06 | 18 | 72661863 | T | A | 0,01114 | -0,408942 | 0,0891385 | NGF | TRUE | reported | ABlBR7 | 5365 | 0,003907713 | 21,03927938 |
| rs180686725 | 1,15758E-06 | 20 | 41625743 | A | G | 0,01335 | -0,404707 | 0,08313 | NGF | TRUE | reported | ABlBR7 | 5365 | 0,004398269 | 23,69212101 |
| rs78398400 | 3,70664E-07 | 1 | 96216680 | A | G | 0,04473 | 0,21409 | 0,0420624 | NDNF | TRUE | reported | qgf5s9 | 5353 | 0,004816269 | 25,89657764 |
| rs34740549 | 4,38258E-06 | 2 | 175626183 | G | T | 0,04058 | 0,205669 | 0,0447386 | NDNF | TRUE | reported | qgf5s9 | 5353 | 0,003932461 | 21,12567534 |
| rs2633260 | 2,65528E-06 | 2 | 233132810 | C | T | 0,1467 | 0,116555 | 0,0247943 | NDNF | TRUE | reported | qgf5s9 | 5353 | 0,004111229 | 22,09000538 |
| rs9883408 | 3,36001E-07 | 3 | 95686751 | C | T | 0,02908 | -0,268085 | 0,0524782 | NDNF | TRUE | reported | qgf5s9 | 5353 | 0,00485152 | 26,08704435 |
| rs190853772 | 2,42226E-06 | 8 | 20775500 | A | C | 0,01211 | -0,378072 | 0,0801052 | NDNF | TRUE | reported | qgf5s9 | 5353 | 0,004144068 | 22,26718508 |
| rs16897941 | 3,96616E-06 | 8 | 124094309 | C | G | 0,2182 | -0,0985448 | 0,0213395 | NDNF | TRUE | reported | qgf5s9 | 5353 | 0,00396803 | 21,31751875 |
| rs11498947 | 1,50702E-06 | 10 | 124048368 | G | C | 0,03032 | 0,24777 | 0,05145 | NDNF | TRUE | reported | qgf5s9 | 5353 | 0,00431372 | 23,18272006 |
| rs150842262 | 9,24209E-07 | 11 | 70843067 | A | G | 0,01291 | -0,385618 | 0,0784911 | NDNF | TRUE | reported | qgf5s9 | 5353 | 0,004488722 | 24,12745267 |
| rs1300085 | 8,92565E-07 | 12 | 52416796 | G | T | 0,08177 | 0,158527 | 0,0322226 | NDNF | TRUE | reported | qgf5s9 | 5353 | 0,004501205 | 24,19485531 |
| rs117820100 | 4,76299E-07 | 12 | 64274147 | T | G | 0,0755 | 0,166802 | 0,0330836 | NDNF | TRUE | reported | qgf5s9 | 5353 | 0,004726311 | 25,41058668 |
| rs704 | 2,37028E-16 | 17 | 26694861 | G | A | 0,4928 | 0,141691 | 0,0172206 | NDNF | TRUE | reported | qgf5s9 | 5353 | 0,012489142 | 67,67459731 |
| rs118162230 | 1,93295E-06 | 22 | 31787595 | C | A | 0,1322 | -0,123658 | 0,0259481 | NDNF | TRUE | reported | qgf5s9 | 5353 | 0,004224715 | 22,70236061 |
| rs3000895 | 0,00000383 | 1 | 12622849 | T | C | 0,936399996 | -0,4222 | 0,09137736 | CNTF | FALSE | reported | lEmqDQ | 997 | 0,020963461 | 21,30527606 |
| rs935486 | 0,00004121 | 2 | 30118859 | A | G | 0,039760001 | -0,4632 | 0,112959384 | CNTF | FALSE | reported | lEmqDQ | 997 | 0,016585701 | 16,78109886 |
| rs716685 | 0,00001629 | 2 | 70793033 | C | T | 0,312099993 | 0,206 | 0,047790493 | CNTF | TRUE | reported | lEmqDQ | 997 | 0,018295201 | 18,54297211 |
| rs4665057 | 0,000009414 | 2 | 159318483 | C | G | 0,281300008 | 0,2222 | 0,0501556 | CNTF | TRUE | reported | lEmqDQ | 997 | 0,019305795 | 19,58741672 |
| rs7565873 | 0,000008937 | 2 | 58815133 | C | T | 0,546700001 | -0,2015 | 0,045368443 | CNTF | FALSE | reported | lEmqDQ | 997 | 0,019401636 | 19,68658012 |
| rs281492 | 0,000008691 | 2 | 46196504 | T | C | 0,504000008 | -0,1991 | 0,044767583 | CNTF | FALSE | reported | lEmqDQ | 997 | 0,019453084 | 19,73981845 |
| rs10866090 | 0,00002614 | 3 | 954702 | G | A | 0,03678 | -0,5253 | 0,124930887 | CNTF | FALSE | reported | lEmqDQ | 997 | 0,017423932 | 17,64424495 |
| rs17028838 | 0,00002088 | 3 | 32081239 | C | T | 0,09245 | 0,3329 | 0,078232465 | CNTF | TRUE | reported | lEmqDQ | 997 | 0,017837813 | 18,07097095 |
| rs10004202 | 0,00001318 | 4 | 171394936 | A | G | 0,184900001 | -0,2551 | 0,058548182 | CNTF | FALSE | reported | lEmqDQ | 997 | 0,018685602 | 18,94619507 |
| rs10041133 | 0,00003078 | 5 | 152949036 | A | G | 0,717700005 | 0,2157 | 0,051756191 | CNTF | TRUE | reported | lEmqDQ | 997 | 0,017122994 | 17,33419217 |
| rs350024 | 0,0000122 | 5 | 40191013 | C | T | 0,042739999 | -0,4496 | 0,102789613 | CNTF | FALSE | reported | lEmqDQ | 997 | 0,018827997 | 19,09334688 |
| rs13212298 | 0,00004461 | 6 | 113921735 | C | T | 0,261400014 | -0,202 | 0,049483035 | CNTF | FALSE | reported | lEmqDQ | 997 | 0,016439776 | 16,63098638 |
| rs10081066 | 0,00001288 | 6 | 155198063 | C | T | 0,658999979 | -0,2069 | 0,047430915 | CNTF | FALSE | reported | lEmqDQ | 997 | 0,018728035 | 18,99004119 |
| rs2390256 | 0,00004442 | 7 | 20138259 | T | C | 0,307200015 | -0,1969 | 0,048221997 | CNTF | FALSE | reported | lEmqDQ | 997 | 0,016447632 | 16,63906672 |
| rs2511585 | 0,00001479 | 8 | 104241486 | A | G | 0,647099972 | 0,2067 | 0,047716962 | CNTF | TRUE | reported | lEmqDQ | 997 | 0,01847321 | 18,72678829 |
| rs10977704 | 0,00001582 | 9 | 9384643 | A | C | 0,267399997 | -0,2285 | 0,052930889 | CNTF | FALSE | reported | lEmqDQ | 997 | 0,018349149 | 18,59867274 |
| rs2039011 | 0,000008048 | 9 | 18956546 | T | G | 0,849900007 | -0,2692 | 0,06030597 | CNTF | FALSE | reported | lEmqDQ | 997 | 0,019594761 | 19,88645745 |
| rs220865 | 0,000007418 | 11 | 115426231 | G | A | 0,779299974 | 0,2211 | 0,049338053 | CNTF | TRUE | reported | lEmqDQ | 997 | 0,019745011 | 20,04201527 |
| rs179095 | 0,00003938 | 14 | 30377669 | A | G | 0,893599987 | 0,3277 | 0,07971127 | CNTF | TRUE | reported | lEmqDQ | 997 | 0,016669319 | 16,86713624 |
| rs2412541 | 0,00003621 | 15 | 40621642 | G | T | 0,85589999 | -0,2492 | 0,060332837 | CNTF | FALSE | reported | lEmqDQ | 997 | 0,016823826 | 17,02615166 |
| rs9941193 | 0,00003978 | 16 | 51755174 | A | G | 0,328000009 | -0,1972 | 0,047995089 | CNTF | FALSE | reported | lEmqDQ | 997 | 0,016650714 | 16,84799188 |
| rs2214346 | 0,000001933 | 16 | 18028711 | T | C | 0,545700014 | -0,2053 | 0,043127483 | CNTF | FALSE | reported | lEmqDQ | 997 | 0,022223583 | 22,61505245 |
| rs8099055 | 0,00002016 | 18 | 8133002 | G | A | 0,01392 | 0,7694 | 0,180478514 | CNTF | TRUE | reported | lEmqDQ | 997 | 0,017902464 | 18,13766071 |
| rs2900918 | 0,00004886 | 19 | 13394519 | C | T | 0,034790002 | 0,4945 | 0,121767612 | CNTF | TRUE | reported | lEmqDQ | 997 | 0,016272293 | 16,45875338 |
| rs199503547 | 0,00000026 | 1 | 199903837 | T | A | 0,1261 | -0,1128 | 0,0219 | GDNF | TRUE | reported | J5jW3a | 10894 | 0,002429329 | 26,52468477 |
| rs7521938 | 0,00000402 | 1 | 237548259 | G | T | 0,0118 | -0,2909 | 0,0631 | GDNF | TRUE | reported | J5jW3a | 11139 | 0,001904385 | 21,24959904 |
| rs144181640 | 0,00000137 | 2 | 207881609 | T | A | 0,0102 | -0,3366 | 0,0697 | GDNF | TRUE | reported | J5jW3a | 13988 | 0,001664499 | 23,31849769 |
| rs62243355 | 0,000000558 | 3 | 36030560 | T | C | 0,0997 | -0,0981 | 0,0196 | GDNF | TRUE | reported | J5jW3a | 14731 | 0,00169768 | 25,04764531 |
| rs531034304 | 0,000000373 | 5 | 36649961 | A | G | 0,0103 | 0,5245 | 0,1032 | GDNF | TRUE | reported | J5jW3a | 8710 | 0,002956837 | 25,82449608 |
| rs11740708 | 7,56E-86 | 5 | 37867986 | A | G | 0,0906 | -0,3888 | 0,0198 | GDNF | TRUE | reported | J5jW3a | 14710 | 0,025543014 | 385,5343517 |
| rs2194229 | 5,34E-21 | 5 | 37918737 | A | T | 0,1296 | -0,1683 | 0,0179 | GDNF | TRUE | reported | J5jW3a | 14288 | 0,006149106 | 88,38964186 |
| rs2453338 | 2,3E-11 | 5 | 38094769 | T | A | 0,648 | -0,0829 | 0,0124 | GDNF | TRUE | reported | J5jW3a | 14734 | 0,003024333 | 44,68962757 |
| rs144945225 | 0,00000252 | 5 | 54809491 | A | C | 0,0155 | 0,3497 | 0,0743 | GDNF | TRUE | reported | J5jW3a | 12005 | 0,001841835 | 22,14834495 |
| rs112594435 | 0,00000427 | 6 | 134367111 | T | C | 0,0371 | 0,1531 | 0,0333 | GDNF | TRUE | reported | J5jW3a | 14721 | 0,001433842 | 21,13503187 |
| rs6461888 | 0,00000172 | 7 | 25845805 | T | C | 0,0131 | 0,4703 | 0,0983 | GDNF | TRUE | reported | J5jW3a | 9054 | 0,002521772 | 22,88479239 |
| rs1362848 | 0,000000837 | 8 | 39994313 | A | G | 0,986 | -0,3148 | 0,0639 | GDNF | TRUE | reported | J5jW3a | 13801 | 0,001755473 | 24,26636858 |
| rs540456481 | 0,0000018 | 8 | 106694458 | T | G | 0,0124 | -0,2941 | 0,0616 | GDNF | TRUE | reported | J5jW3a | 10894 | 0,002088014 | 22,79023936 |
| rs2093446 | 0,00000263 | 9 | 5230371 | C | G | 0,3 | 0,0606 | 0,0129 | GDNF | TRUE | reported | J5jW3a | 14288 | 0,001542141 | 22,0650559 |
| rs77671113 | 9,45E-09 | 10 | 85934651 | A | C | 0,0859 | -0,1194 | 0,0208 | GDNF | TRUE | reported | J5jW3a | 14725 | 0,002232831 | 32,94753988 |
| rs138691533 | 0,00000163 | 12 | 98235676 | A | G | 0,0148 | 0,2584 | 0,0539 | GDNF | TRUE | reported | J5jW3a | 14731 | 0,001557752 | 22,97992045 |
| rs57104839 | 0,00000304 | 13 | 27573222 | G | A | 0,097 | -0,0915 | 0,0196 | GDNF | TRUE | reported | J5jW3a | 14736 | 0,001476756 | 21,79069581 |
| rs79953230 | 0,00000496 | 13 | 66719542 | T | C | 0,0254 | 0,1918 | 0,042 | GDNF | TRUE | reported | J5jW3a | 14719 | 0,001414834 | 20,85161077 |
| rs35740453 | 0,00000313 | 17 | 1587488 | G | A | 0,2627 | 0,0718 | 0,0154 | GDNF | TRUE | reported | J5jW3a | 13224 | 0,001641086 | 21,73410491 |
| rs9303102 | 0,00000371 | 17 | 14317973 | T | G | 0,0231 | 0,2096 | 0,0453 | GDNF | TRUE | reported | J5jW3a | 12935 | 0,001652348 | 21,40518556 |
| rs55850217 | 5,59E-09 | 19 | 14694468 | T | C | 0,3119 | 0,0816 | 0,014 | GDNF | TRUE | reported | J5jW3a | 11755 | 0,002881697 | 33,96646485 |
| rs570043519 | 0,0000041 | 21 | 18870063 | A | T | 0,02 | -0,3321 | 0,0721 | GDNF | TRUE | reported | J5jW3a | 8911 | 0,002375243 | 21,21141969 |
| rs145686993 | 0,00000484 | 2 | 46079035 | C | G | 0,0161 | -0,2464 | 0,0539 | NT-3 | TRUE | reported | oeKFMI | 14257 | 0,001463658 | 20,89502758 |
| rs79759094 | 0,0000036 | 4 | 7531372 | A | G | 0,0638 | -0,1163 | 0,0251 | NT-3 | TRUE | reported | oeKFMI | 14728 | 0,001455578 | 21,46609303 |
| rs12517801 | 0,0000045 | 5 | 1730939 | A | G | 0,3609 | -0,0555 | 0,0121 | NT-3 | TRUE | reported | oeKFMI | 14726 | 0,001426627 | 21,03566463 |
| rs2034246 | 0,00000123 | 5 | 115115890 | C | A | 0,4323 | -0,0582 | 0,012 | NT-3 | TRUE | reported | oeKFMI | 14296 | 0,001642687 | 23,51920922 |
| rs3218587 | 0,00000349 | 6 | 111367724 | A | G | 0,0429 | 0,1364 | 0,0294 | NT-3 | TRUE | reported | oeKFMI | 14744 | 0,001457757 | 21,52163019 |
| rs112418830 | 0,000000206 | 6 | 150972752 | T | C | 0,0567 | 0,1366 | 0,0263 | NT-3 | TRUE | reported | oeKFMI | 14296 | 0,001883461 | 26,97299302 |
| rs2107473 | 0,00000236 | 7 | 8723326 | G | A | 0,9825 | 0,2393 | 0,0507 | NT-3 | TRUE | reported | oeKFMI | 13394 | 0,001660494 | 22,27432872 |
| rs17537796 | 0,00000333 | 7 | 39986573 | C | A | 0,0134 | 0,2571 | 0,0553 | NT-3 | TRUE | reported | oeKFMI | 14254 | 0,001514116 | 21,61190003 |
| rs73166403 | 0,00000422 | 7 | 152601143 | T | C | 0,1114 | -0,092 | 0,02 | NT-3 | TRUE | reported | oeKFMI | 14296 | 0,001477947 | 21,15703973 |
| rs72652073 | 0,00000348 | 8 | 71119666 | C | G | 0,3811 | 0,058 | 0,0125 | NT-3 | TRUE | reported | oeKFMI | 14296 | 0,001503723 | 21,52658802 |
| rs10780690 | 0,00000346 | 9 | 84863257 | G | T | 0,4735 | -0,0557 | 0,012 | NT-3 | TRUE | reported | oeKFMI | 14296 | 0,001504802 | 21,5420553 |
| rs146532158 | 0,00000146 | 10 | 114074103 | T | C | 0,0249 | -0,1859 | 0,0386 | NT-3 | TRUE | reported | oeKFMI | 14741 | 0,001570994 | 23,19130795 |
| rs138731987 | 0,00000327 | 11 | 93231828 | T | C | 0,0283 | 0,2806 | 0,0603 | NT-3 | TRUE | reported | oeKFMI | 12156 | 0,001778186 | 21,65056577 |
| rs11225042 | 0,00000164 | 11 | 101856953 | C | A | 0,1263 | 0,0973 | 0,0203 | NT-3 | TRUE | reported | oeKFMI | 12412 | 0,001847518 | 22,97013879 |
| rs2846299 | 0,00000322 | 11 | 123504348 | G | A | 0,6978 | -0,0582 | 0,0125 | NT-3 | TRUE | reported | oeKFMI | 14721 | 0,001470448 | 21,67539077 |
| rs58399805 | 0,000000498 | 12 | 5570938 | A | T | 0,0799 | 0,1106 | 0,022 | NT-3 | TRUE | reported | oeKFMI | 14737 | 0,001712031 | 25,27004114 |
| rs540074689 | 0,000000896 | 14 | 18580280 | C | A | 0,1996 | 0,1076 | 0,0219 | NT-3 | TRUE | reported | oeKFMI | 10703 | 0,002250362 | 24,13543617 |
| rs28735437 | 1,06E-12 | 15 | 87971624 | C | G | 0,1435 | -0,1168 | 0,0164 | NT-3 | TRUE | reported | oeKFMI | 14737 | 0,00343002 | 50,71530552 |
| rs187443426 | 0,00000467 | 17 | 31516672 | G | A | 0,0132 | 0,2752 | 0,0601 | NT-3 | TRUE | reported | oeKFMI | 13809 | 0,001516096 | 20,9645242 |
| rs376150779 | 0,00000192 | 18 | 57642828 | G | C | 0,2265 | 0,0819 | 0,0172 | NT-3 | TRUE | reported | oeKFMI | 10894 | 0,002076924 | 22,66893783 |
| rs11151953 | 3,23E-09 | 18 | 74456625 | C | G | 0,5258 | 0,0734 | 0,0124 | NT-3 | TRUE | reported | oeKFMI | 14296 | 0,002444956 | 35,03385981 |
| rs5751454 | 0,00000464 | 22 | 43253709 | T | C | 0,0287 | 0,1823 | 0,0398 | NT-3 | TRUE | reported | oeKFMI | 13680 | 0,001531283 | 20,97701531 |
| rs17320368 | 0,00004752 | 1 | 63743494 | C | G | 0,047710001 | -0,3808 | 0,093620136 | NT-4 | FALSE | reported | 2bvlX2 | 997 | 0,01632347 | 16,51137588 |
| rs10752934 | 0,00003285 | 1 | 184199676 | A | G | 0,335000008 | 0,1986 | 0,047823703 | NT-4 | FALSE | reported | 2bvlX2 | 997 | 0,017003137 | 17,21075866 |
| rs17086570 | 0,0000109 | 6 | 155678361 | G | A | 0,079520002 | -0,3438 | 0,078162953 | NT-4 | FALSE | reported | 2bvlX2 | 997 | 0,019035658 | 19,30802066 |
| rs11765423 | 0,000001789 | 7 | 96560195 | A | G | 0,170000002 | -0,2748 | 0,057538822 | NT-4 | FALSE | reported | 2bvlX2 | 997 | 0,022366219 | 22,76352178 |
| rs11198333 | 0,00001942 | 10 | 83368626 | G | A | 0,234599993 | 0,226 | 0,052909347 | NT-4 | FALSE | reported | 2bvlX2 | 997 | 0,017971365 | 18,20874418 |
| rs34154656 | 0,0000411 | 11 | 56629040 | G | A | 0,045729998 | -0,5027 | 0,12257366 | NT-4 | FALSE | reported | 2bvlX2 | 997 | 0,016590621 | 16,78616099 |
| rs11109941 | 0,00001999 | 12 | 99601889 | A | G | 0,163000003 | 0,2568 | 0,060210985 | NT-4 | FALSE | reported | 2bvlX2 | 997 | 0,017918066 | 18,15375602 |
| rs423694 | 0,00004892 | 16 | 83839259 | C | T | 0,362800002 | 0,1912 | 0,047085157 | NT-4 | FALSE | reported | 2bvlX2 | 997 | 0,016270035 | 16,45643122 |
| rs9922084 | 0,00003829 | 16 | 19658148 | G | T | 0,066600002 | 0,3859 | 0,093720486 | NT-4 | FALSE | reported | 2bvlX2 | 997 | 0,016720994 | 16,92031377 |
| rs6030585 | 0,00001985 | 20 | 42985066 | C | G | 0,804199994 | -0,2418 | 0,056673141 | NT-4 | FALSE | reported | 2bvlX2 | 997 | 0,017931015 | 18,16711456 |
